# Cellular levels and molecular dynamics simulations of estragole DNA adducts point at inefficient repair resulting from limited distortion of the double-stranded DNA helix

**DOI:** 10.1007/s00204-020-02695-5

**Published:** 2020-03-18

**Authors:** Shuo Yang, Matthias Diem, Jakob D. H. Liu, Sebastiaan Wesseling, Jacques Vervoort, Chris Oostenbrink, Ivonne M. C. M. Rietjens

**Affiliations:** 1 Division of Toxicology, Wageningen University, Stippeneng 4, 6708 WE Wageningen, The Netherlands; 2 Department of Material Sciences and Process Engineering, Institute of Molecular Modeling and Simulation, University of Natural Resources and Life Science, Vienna, Austria; 3 Division of Biochemistry, Wageningen University, Stippeneng 4, 6708 WE Wageningen, The Netherlands

**Keywords:** Estragole, DNA adduct, DNA repair efficiency, Molecular modeling and simulation

## Abstract

Estragole, naturally occurring in a variety of herbs and spices, can form DNA adducts after bioactivation. Estragole DNA adduct formation and repair was studied in in vitro liver cell models, and a molecular dynamics simulation was used to investigate the conformation dependent (in)efficiency of *N*^2^-(*trans*-isoestragol-3′-yl)-2′-deoxyguanosine (E-3′-*N*^2^-dG) DNA adduct repair. HepG2, HepaRG cells, primary rat hepatocytes and CHO cells (including CHO wild-type and three NER-deficient mutants) were exposed to 50 μM estragole or 1′-hydroxyestragole and DNA adduct formation was quantified by LC–MS immediately following exposure and after a period of repair. Results obtained from CHO cell lines indicated that NER plays a role in repair of E-3′-*N*^2^-dG adducts, however, with limited efficiency since in the CHO wt cells 80% DNA adducts remained upon 24 h repair. Inefficiency of DNA repair was also found in HepaRG cells and primary rat hepatocytes. Changes in DNA structure resulting from E-3′-*N*^2^-dG adduct formation were investigated by molecular dynamics simulations. Results from molecular dynamics simulations revealed that conformational changes in double-stranded DNA by E-3′-*N*^2^-dG adduct formation are small, providing a possible explanation for the restrained repair, which may require larger distortions in the DNA structure. NER-mediated enzymatic repair of E-3′-*N*^2^-dG DNA adducts upon exposure to estragole will be limited, providing opportunities for accumulation of damage upon repeated daily exposure. The inability of this enzymatic repair is likely due to a limited distortion of the DNA double-stranded helix resulting in inefficient activation of nucleotide excision repair.

**Electronic supplementary material:**

The online version of this article (10.1007/s00204-020-02695-5) contains supplementary material, which is available to authorized users.

## Introduction

**Fig. 1:**
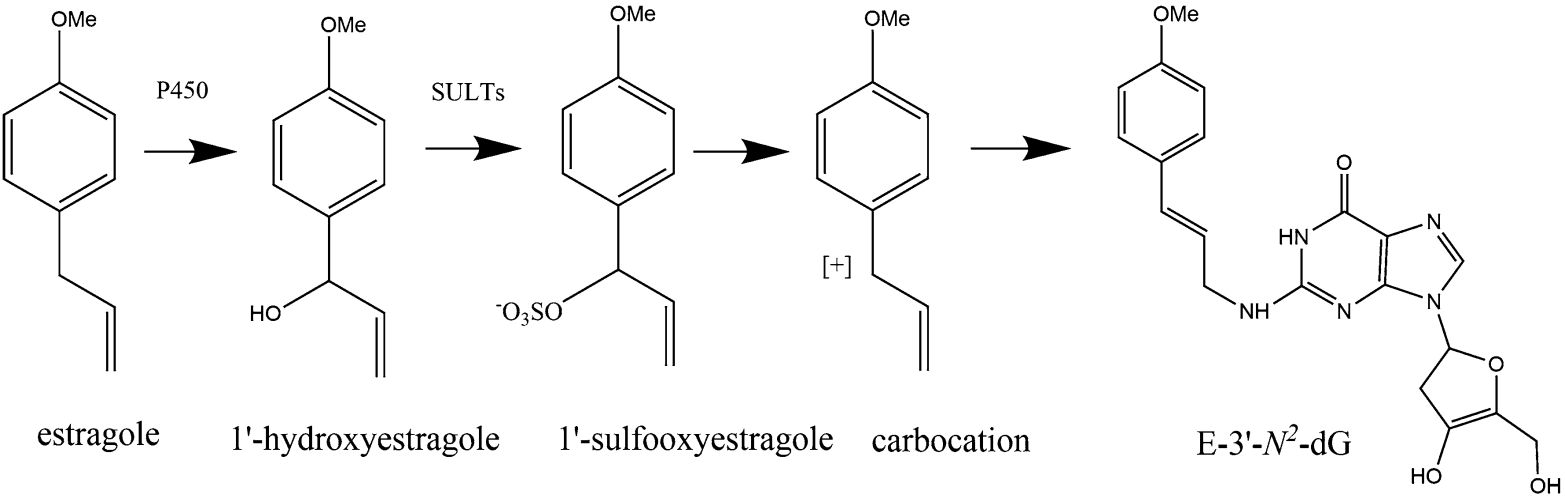
Metabolic pathway for bioactivation of estragole and *N*^2^-(*trans*-isoestragol-3′-yl)-2′-deoxyguanosine (E-3′-*N*^2^-dG) adduct formation

Estragole, one of the food-borne alkenylbenzenes, can naturally occur in a variety of herbs and spices such as sweet basil, fennel, star anise, and essential oils (Rietjens et al. 2014). Upon dietary intake, estragole can be rapidly absorbed by the gastrointestinal tract and bioactivated in the liver (Smith et al. 2002). An overview of the bioactivation pathway of estragole is presented in Fig. 1. Briefly, the bioactivation proceeds by cytochrome P450 catalyzed formation of the proximate carcinogen 1′-hydroxyestragole (1′-OH estragole), upon which 1′-OH estragole is sulfonated by sulfotransferases (SULTs) to produce the ultimate carcinogenic metabolite 1′-sulfoxyestragole that can result in DNA adduct formation and contributes to the induction of hepatocarcinogenicity (Paini et al. 2010). The major DNA adduct formed is *N*^2^-(*trans*-isoestragol-3′-yl)-2′-deoxyguanosine (E-3′-*N*^2^-dG) (Fig. 1) (Phillips et al. 1981; Punt et al. 2007).

When considering estragole DNA adduct formation it is important to note that the level of DNA adducts depends on not only their formation via the reactive 1′-sulfooxy metabolite but also on the efficiency of their possible repair. In mammalian cells, bulky DNA lesions can be repaired by the nucleotide excision repair (NER) mechanism. In this mechanism a set of enzymes including XPC-RAD23B recognizes the local distortion and continues to recruit other factors like TFIIH, XPB, XPD, etc. to initiate the process of repair (Schärer 2013). Some bulky DNA adducts, such as adducts formed by aristolochic acids, aflatoxin B1, and benzo(a)pyrene have, however, been reported to merge with the DNA double-stranded helix in such a way that they do not result in a significant disturbance of the overall DNA structure, resulting in these adducts being relatively resistant to NER (Geacintov and Broyde 2017). A recent study did report the detection of *N*^6^-(methylisoeugenol-3′-yl)-2′-deoxyadenosine (ME-dA) in the urine of rats exposed to different plant extractions containing the related alkenylbenzene methyleugenol for 12 h (Feng et al. 2018). The occurrence of this DNA adduct in the urine may reflect direct interaction of the reactive intermediate with free dA but may also in part result from NER mediated repair of ME DNA adducts as suggested by Feng et al. (2018). On the other hand, Herrmann et al. (2013) reported detection of methyleugenol DNA adducts in the livers of Caucasian subjects. This revealed that repair may be not fully efficient and/or that the DNA repair is not sufficient to balance DNA formation as a result of exposure to methyleugenol from a regular diet, resulting in detectable levels of adducts. The efficiency of repair of alkenylbenzene DNA adducts has, however, not been studied in detail so far. Therefore, the aim of the present paper is to quantify DNA adduct formation and repair of the alkenylbenzene estragole in different in vitro cell models and study how this adduct formation impacts the conformation of the double-strand DNA helix using molecular modelling.

## Material and method

### Chemicals and reagents

Estragole, human insulin, ammonium bicarbonate, dexamethasone (DEX), bovine spleen phosphodiesterase II (SPDE II), venom phosphodiesterase I (VPDE I), nuclease P1, phosphatase alkaline (AP), 3-(4,5-dimethyl-2-thiazolyl)-2,5-diphenyl-2*H*-tetrazolium bromide (MTT), tris(hydroxymethyl)aminomethane (Tris), ethylenediaminetetraacetic acid (EDTA), and 2′-deoxyguanosine were obtained from Sigma (St. Louis, Missouri, USA). Minimum Essential Medium (MEM), l-glutamine, Dulbecco’s Modified Eagle Medium: Nutrient Mixture F-12 (DMEM/F-12), alpha minimum essential media (αMEM), trypsin, and phosphate-buffered saline (PBS) (pH 7.4) were purchased from Gibco (Paisley, UK). Non-Essential Amino Acids (NEAA), Williams E Medium and penicillin–streptomycin (P/S) were purchased from Gibco (Grand Island, NY, USA). CM3000 and 4000 kits were purchased from Gibco (Frederick, MD, USA). Fetal Bovine Serum (FBS) was purchased from Bodinco BV (Alkmaar, Netherlands). Hydrocortisone 21-hemisuccinate was purchased from Cayman Chemical (Ann Arbor, MI, USA). Dimethylsulfoxide (DMSO), hydrochloric acid (HCl), zinc sulfate (ZnSO_4_), sodium acetate, and acetic anhydride were purchased from Merck (Darmstadt, Germany). 4-[3-(4-Iodophenyl)-2-(4-nitrophenyl)-2*H*-5-tetrazolio]-1,3-benzene disulfonate (WST-1) was obtained from Roche (Mannheim, Germany). Acetonitrile (ACN) was purchased from Biosolve (Dieuze, France). RLT lysis buffer was purchased from QIAGEN (Hilden, Germany). Formic acid was purchased from VMR (Fontenay-sous-Bois, France). Beta-naphthoflavone (BNF) was purchased from Fluka Chemie GmbH (Buchs, Switzerland). 1′-OH estragole was synthesized as described previously (Paini et al. 2010).

### Cellular models

The human hepatoma cell line HepG2 cells were obtained from the American Type Culture Collection (Manassas, VA, USA). Cells were cultured in MEM containing Earle’s Salts, supplemented with 10% (*v*/*v*) FBS, 1% (*v*/*v*) P/S, 1% (*v*/*v*) NEAA, and 2 mM l-glutamine. Cells were incubated at 37 °C with 5% CO_2_ and subcultured every 3 or 4 days.

The human hepatoma cell line HepaRG (undifferentiated cells) was purchased from Biopredic International (Saint Grégoire, France). The cells were first incubated in growth medium containing Williams E Medium containing 10% FBS (Sigma, St. Louis, MO, USA), 100 IU/ml of P/S, 5 × 10^−5^ M hydrocortisone 21-hemisuccinate and 5 μg/ml human insulin for 2 weeks. In the following 2 weeks, differentiation medium was used to facilitate the differentiation of the cells into cells with hepatocyte-like morphology after which the cells were cultured for another 2 weeks in the same medium supplemented with 1.7% DMSO to obtain maximum differentiation. HepaRG cells plated in 96-well plates (Greiner Bio-One, Frickenhausen, Germany) and T-25 flasks (Greiner Bio-One, Frickenhausen, Germany) were used for the cytotoxicity tests and in vitro DNA adduct formation experiments, respectively.

Rat (Wistar) primary hepatocytes in suspension (cryopreserved, male) were purchased from Thermo Fisher Scientific (Bleiswijk, Netherlands). The CM3000 kit was used for thawing the cells and the CM4000 kit was used for cell maintenance. All the kits were dissolved in Williams E Medium without phenol red according to the protocol provided by Thermo Fisher Scientific.

Chinese Hamster Ovary (CHO) wild-type and UV-mutated types (UV5, UV41 and UV24), in which NER activity is absent, were obtained from ATCC (Manassas, VA, USA). Wild-type cells were grown in DMEM/F-12 containing 10% (*v*/*v*) FBS and 1% (*v*/*v*) P/S. Mutant cells were grown in αMEM with 10% (*v*/*v*) FBS. All cells were incubated at 37 °C with 5% (*v*/*v*) CO_2_ until reaching 80% confluence.

### Cytotoxicity test

Cytotoxicity was tested by the MTT assay (HepG2 and HepaRG cells) or the WST assay (hepatocytes). To this end, HepG2 cells, HepaRG cells, and rat hepatocytes were seeded in 96-well plates at the concentration of 2 × 10^5^ cells/ml, 0.9 × 10^5^ cells/ml, and 5 × 10^5^ cells/ml, respectively. The cells were exposed to serum-free medium containing (final concentration) 50 μM estragole or 1′-OH estragole with 0.1% DMSO for 2 h. Each compound was tested in three independent experiments. After exposure, 10 μl 5 mg/ml MTT for HepG2 and HepaRG cells, or 10 μl WST-1 reagent for rat hepatocytes were added to each well followed by incubation for another hour. For the MTT assay, the medium was removed and 100 μl of DMSO was added to the wells to dissolve the MTT formazan crystals. For the WST-1 assay, no additional handlings were required. The absorbance was measured at 562 nm for the MTT assay and at 440 nm for the WST assay using a SpectraMax M2 (Molecular Devices, USA). The cell viability was expressed as % of the control, with the solvent control set at 100% viability.

### In vitro DNA adduct formation

#### Induction of cytochromes P450

To increase cellular levels of cytochromes P450 required for estragole bioactivation to 1′-OH estragole, HepG2 cells and HepaRG cells were cultured in 25 cm^2^ flasks and incubated at 37 °C and 5% (*v*/*v*) CO_2_ in a humidified atmosphere in the presence of inducers of cytochrome P450 1A2 and 2A6 the isoenzymes previously shown to be involved in estragole 1′-hydroxylation (Jeurissen et al. 2007). Once 60–70% confluence was reached, HepG2 cells were maintained in medium with reduced FBS (2%) for 2 h after which the cells were incubated with the CYP1A2 inducer BNF (final concentration 5 μM) or the CYP2A6 inducer DEX (final concentration 50 μM) in the same medium for 3 days, with medium renewal every 24 h.

Induction for differentiated HepaRG cells was started in the first week after full differentiation. To this end the differentiation medium was changed to growth medium and cells were cultured in this medium for another 3 days. After that, the induction medium (Biopredic) with 2% FBS containing BNF (final concentration 5 or 25 μM) or DEX (final concentration 50 μM) was applied for another 3 days with medium renewal every 24 h.

Stock solutions of inducers were prepared in DMSO and further diluted with induction medium to give a final concentration of DMSO of 0.1%. Control cells (non-induced) were exposed to 0.1% (*v*/*v*) DMSO during the induction period.

#### Exposure of cellular models to estragole and 1′-OH estragole

HepG2 cells and HepaRG cells including induced and non-induced cells, and non-induced primary rat hepatocytes were exposed to estragole or 1′-OH estragole at a final concentration of 50 μM for 2 h. The test compounds were dissolved in DMSO and then diluted in exposure medium, with 0.1% final concentration of DMSO. After 2 h of incubation, cells were washed with PBS. HepG2 cells and HepaRG cells were detached by using trypsin and collected in PBS. For primary rat hepatocytes, cells were kept in suspension in 6-well plates (Corning, Kennebunk, USA) by gentle shaking at a density of 1 × 10^6^ cells/well. After exposure, the cells were centrifuged at 500 rpm (Hermle Z400K Refrigerated Centrifuge, Germany) for 5 min to discard the exposure medium and the cell pellet was washed with PBS. All cells were collected in 1 ml PBS in the Eppendorf tube and centrifuged at 1500 rpm for 5 min. The washing and centrifugation step was repeated one more time, and the pellet was lysed into 200 μl RLT lysis buffer before DNA isolation.

#### DNA isolation and digestion

DNA was isolated using the QIAamp DNA Mini Kit protocol for cultured cells (Hilden, Germany). The number of cells for each sample was between 2 × 10^6^ and 5 × 10^6^ cells. The yield and purity of the isolated DNA were determined by Nanodrop 2000 technology (Thermo Scientific, Wilmington, DE USA) measuring the absorbance ratio A260/280 nm. DNA samples with a ratio of 1.8–2.0 were considered sufficiently pure. After quantifying the DNA, samples were freeze-dried and nanopure water was added to the samples to obtain the final amount of 50 μg DNA in 30 μl water. DNA digestion was applied as previously described by Paini et al. (2010) with minor adjustments. In short, 40 μl PI-buffer (300 mM sodium acetate, 1 mM ZnSO_4_, pH 5.3), 20 μl SPDE II solution (0.0004 U/μl in water), and 10 μl nuclease P1 (0.5 μg/μl in water) was added to DNA samples and then the samples were incubated at 37 °C for 4 h. Then the samples were incubated with a mixture of 40 μl PA-buffer (500 mM Tris–HCl, 1 mM EDTA, pH 8.0), 20 μl VPDE I solution (0.00026 U/μl in water), and 1.6 μl AP (200 units) for another 2 h. After incubation, samples were freeze-dried and stored at − 80 °C until LC–MS/MS analysis. Before analysis the dry, digested samples were dissolved in 25 μl ultrapure water for E-3′-*N*^2^-dG detection.

### DNA repair assay

The role of NER in the repair of E-3′-*N*^2^-dG DNA adducts was investigated in CHO wild-type cells and three NER deficient mutants. Although the CHO cells are ovary instead of liver cells, the set of wild-type and NER-deficient mutants presents a representative model to study the role of human NER since the CHO cells have been shown to contain the human genes involved in NER (Rolig et al. 1998). Given that CHO cells do not contain cytochromes, P450 needed for bioactivation of estragole, estragole DNA adduct repair in these cells was studied upon generation of the DNA adducts via exposure of the cells to 1′-OH estragole. The concentration of 1′-OH estragole used was adapted to result in DNA adduct levels that amounted to levels comparable to what was observed in primary hepatocytes and HepaRG cells in order not to overload the NER system. To this end CHO wild-type and UV-mutated CHO cells were exposed to 50 μM 1′-OH estragole for 2 h at 37 °C to generate DNA adducts at levels < 50/10^8^ nts. The DNA repair assay was additionally performed in non-induced HepaRG cells and rat hepatocytes which were exposed to 50 μM estragole for 2 h. After exposure, the cells were washed with PBS once, the medium was replaced with fresh medium without the test compound, and cells were incubated for 0 h, 2 h, 4 h, 24 h, 48 h, and 72 h before they were harvested for measurement of (residual) DNA adduct levels.

### Synthesis of E-3′-*N*^2^-dG adduct

DNA adducts were synthesized via the reaction of 1′-acetoxyestragole with 2′-deoxyguanosine following the protocol described by Punt et al. (2007). Synthesis of 1′-acetoxyestragole was based on Paini et al. (2010) with the slight modification of using 110 μl of acetic anhydride instead of 35 μl. For E-3′-*N*^2^-dG adduct synthesis; in short, 250 μl of 1′-acetoxyestragole in DMSO (0.01 g/ml) was mixed with 2250 μl of 2.5 mM 2′-deoxyguanosine in 2.5 mM ammonium bicarbonate (pH 7.4). The incubation was stirred over the weekend at 37 °C. The DNA adducts were purified using a Waters Agilent HPLC (Etten-Leur, Netherlands) on an Alltima C18 5 μm column, 150 × 4.6 mm (Alltech, Breda, Netherlands). The gradients were made with nanopure water as solvent A and ACN as solvent B. The flow rate was 1 ml/min. The start condition was 80/20 (A/B), changing to 70/30 (A/B) from 0 to 40 min after which the percentage of ACN increased to 100% over 2 min and was kept at 100% for 1 min. Then the gradient changed back to the start condition in 2 min and was kept at that condition for 15 min. Detection was carried out using a photodiode array detector (Waters, Milford, MA, USA) at 260 nm. E-3′-*N*^2^-dG eluted at 16.80 min. Peaks of several injections were collected and combined, and freeze dried. The purified adduct thus obtained was weighted and then used in LC–MS/MS to establish calibration curves for the quantification of DNA adducts isolated from exposed cells.

### LC–MS/MS method for detection and quantification of E-3′-*N*^2^-dG adducts

The LC–MS/MS method for detection of the E-3′-*N*^2^-dG adducts was adapted from Paini et al. (2010). The analysis was performed on a Shimadzu Nexera XR LC-20AD SR UPLC system coupled with a Shimadzu LCMS-8040 mass spectrometer (Kyoto, Japan). 5 μl of the sample from digested HepG2 cell DNA, HepaRG cell DNA, rat hepatocyte DNA or CHO cell DNA were injected into the 1.7 μm 50 × 2.1 mm column (Phenomenex, California, USA) at 40 °C. The gradient was made with ultra-pure water (solvent A) and ACN (solvent B). Both solvents contained 0.1% (*v*/*v*) formic acid. The flow rate was 0.3 ml/min and each run was 10 min in total. The initial condition was 95/5 (A/B) for 1 min; a linear gradient was applied from 5 to 100% acetonitrile over 8 min and maintained for 0.5 min. Then the gradient returned to the start condition in 0.1 min and was kept for the remaining time. E-3-*N*^2^-dG eluted at 5.99 min. The MS–MS analysis was carried out using a Shimadzu LCMS-8040 triple quadrupole with electrospray ionization (ESI) interface. The instrument was operated in positive mode in the multiple reaction monitoring (MRM) mode with a spray voltage of 4.5 kV. The transitions (*m*/*z*) used for obtaining the daughter fragments were 414.2→298.2, 414.2→164.1, and 414.2→147 for E-3′-*N*^2^-dG. Calibration curves were established by plotting the peak area of a known concentration of the synthesized DNA adduct against the corresponding DNA adduct concentrations. The amount of the DNA adducts detected in the samples was related to the total amount of digested DNA detected in each sample and expressed as the number of E-3′-*N*^2^-dG adducts per 10^8^ nucleotides (nts) based on the assumption of 1.98 × 10^15^ nts/μg DNA.

### Molecular modelling

#### Starting structure and force field

**Fig. 2:**
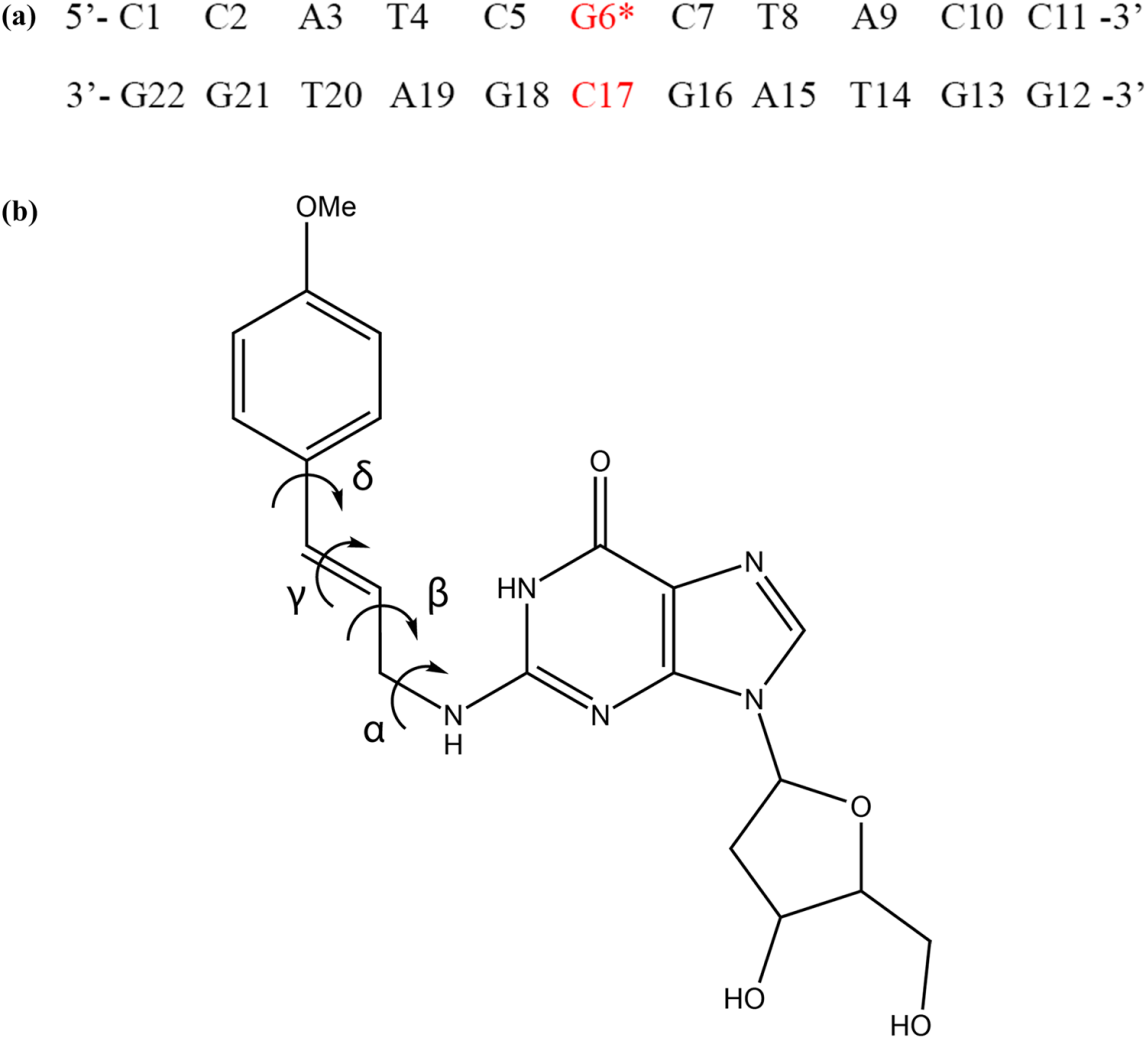
**a** Base sequence context in which the adduct is embedded. G6* represents the modified guanine base and **b** chemical structure of the E-3′-*N*^2^-dG adduct with dihedral angles *α*, *β*, *γ,* and *δ*

Since no experimentally determined structure of E-3′-*N*^2^-dG is available, the Molecular Operating Environment (MOE) software (version 2018.0101, Chemical Computing Group, Montreal) was employed to build an initial model for E-3′-*N*^2^-dG directly on the G6* of a 11-mer base sequence of B-type form DNA (Fig. 2a). The DNA sequence chosen in the present study is the same as the one used previously to study ( +)-*cis*-B[a]PDE-*N*^2^-dG or (–)-*trans*-B[a]PDE-*N*^2^-dG enabling comparison of the results (Mocquet et al. 2007). All molecular interactions were described by the GROMOS force field, parameter set 45A4 (Soares et al. 2005). Corresponding parameters were assigned to the modified base, as described in Supplementary information Fig. S1. Both reference DNA and modified DNA (with adduct) were solvated in a periodic rectangular box with a 1.4-nm minimum distance for solutes to box edges and a 0.23-nm minimum distance for the solute to solvent, containing explicit *simple point charge* (SPC) water and neutralized with 20 Na^+^ counterions. The concentration of additional sodium chloride was 0.2 M. Before the simulation, the models were energy minimized by the steepest descent algorithm to optimize the geometry. A systematic search was performed on the dihedral angles *α*, *β*, *γ,* and *δ* (Fig. 2b) and diverse low-energy conformations were selected. The additional information of selected conformations are described in detail in Results section. For the unmodified reference simulations, four independent simulations were performed.

#### Molecular dynamics simulations

All the simulations were performed using the GROMOS11 molecular simulation package (Version 1.5.0; Biomos B.V.) (Schmid et al. 2012). Bond-length constraints of solute and solvent were imposed by the SHAKE algorithm with a 2-fs time step. A pairlist was generated every five steps using a grid algorithm (Heinz and Hünenberger 2004). The short-range cut-off interactions up to a distance of 0.8 nm were computed at every timestep and intermediate-range interactions up to 1.4 nm were computed at pairlist construction and kept constant in between. Long-range interactions were approximated by a reaction-field contribution, assuming a homogenous medium with a dielectric permittivity of 61 beyond a distance of 1.4 nm (Heinz et al. 2001; Tironi et al. 1995). Prior to equilibration, initial velocities were sampled from a Maxwell–Boltzmann distribution at 60 K. The system was gradually heated up to 300 K in five equidistant steps. In order to restrain the solute position during equilibration, a harmonic potential-energy term was used. The force constant for solute position restraints was decreased by a factor of ten at each equilibration step with an initial value of 2.5 × 10^4^ kJ/mol/nm^2^. In addition, the first and last GC base pairs were kept together by applying half harmonic attractive distance restraints on the hydrogen bonds to avoid the disruption of the initial conformation during the molecular dynamics (MD) simulation. After equilibration of 0.5 ns, constant temperature and pressure were imposed for the subsequent simulation. The temperature remained at 300 K via weak coupling, employing two separate temperature baths for solute and solvent with 0.1 ps coupling time. Additionally, the pressure was set to 1 atm with an isothermal compressibility of 4.575 × 10^–4^ (kJ mol^–1^ nm^–3^)^–1^ and a coupling time of 0.5 ps (Berendsen et al. 1984). Overall center of mass motion was removed every 2 ps. The simulations were performed for 20 ns.

#### Structural analysis

The GROMOS++ programs were employed for structural analysis (Eichenberger et al. 2011). A conformational clustering was conducted on the conformation of the adduct, after a superposition of the DNA backbones. For this, structures, separated by 5 ps were collected from all four adduct simulations and the atom-positional root-mean-square deviation (RMSD) were calculated between all pairs of structures. Structures within 0.2 nm of each other were considered structural neighbors and the clustering algorithm described by Daura et al. (1999) was used. For each of the clusters, the central member structure was selected as a representative structure. The stability of the MD simulation for the reference double-stranded DNA helix and the modified DNA helix (with DNA adduct) was tested by monitoring RMSD, which represent the deviations in each trajectory snapshot relative to the respective starting structure. The form of the DNA helix was classified by the DISICL algorithm (Nagy and Oostenbrink 2014). The hydrogen bonding occupancy was analyzed using a geometric criterion. A hydrogen bond was considered to be formed when the hydrogen-acceptor distance was within 0.25 nm and the donor-hydrogen-acceptor angle was larger than 135°. The total non-bonded interaction energy for the whole DNA structure and van der Waals interactions between the adduct and the rest of the residues without solvent were calculated. DNA duplex helicoidal parameters and groove dimensions were analyzed over the whole trajectories by X3DNA (Lu and Olson 2003). For this GROMOS trajectories were converted to PDB files and parsed individually. PyMOL was used to make molecular images and movies (DeLano 2002), (Version 2.3, 2019).

### Statistical analysis

Durnnett’s Multiple Comparison Test was performed using GraphPad Prism 5 (Version 5.04, 2010; GraphPad Software, Inc).

## Results

### Cytotoxicity test

The test compounds estragole and 1′-OH estragole were not cytotoxic to the HepG2 cells, HepaRG cells or primary rat hepatocytes at concentrations applied in the experiments as detected in the MTT or WST-1 assay (data not shown).

### DNA adduct formation in non-induced liver cell models

**Fig. 3:**
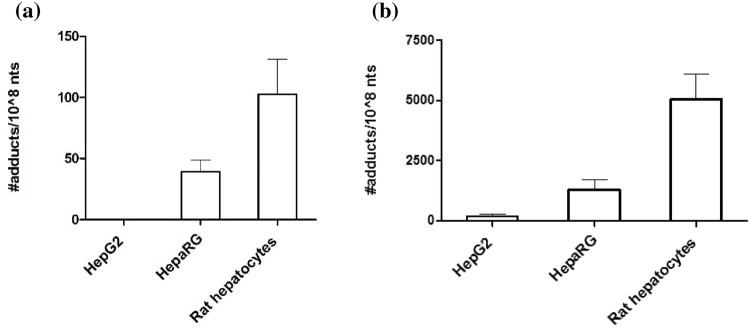
E-3′-*N*^2^-dG formation in non-induced HepG2 cells, HepaRG cells, and primary rat hepatocytes exposed to **a** 50 μM estragole and **b** 50 μM 1′-OH estragole for 2 h. Data represent the mean (+ SD) from three independent experiments. Note the 50-fold different scale of the Y-axis in figure **a** and **b**

In order to select the most suitable liver cell model for subsequent DNA repair experiments, studies of E-3′-*N*^2^-dG formation in HepG2 cells, HepaRG cells, and primary rat hepatocytes were performed. To this end cells were exposed to both the parent compound estragole and the proximate carcinogenic metabolite 1′-OH-estragole. The number of adducts measured after exposure to 50 μM estragole or 1′-OH estragole in the different cell models is shown in Fig. 3. The data reveal that in all cell models DNA adduct formation is readily detectable upon exposure to 1′-OH estragole with the amount increasing in the order HepG2 cells < HepaRG cells < primary rat hepatocytes (Fig. 3b). Upon exposure of the cells to estragole, DNA adduct formation was only detected in HepaRG and rat hepatocytes albeit at levels that were, respectively, 33- and 40-fold lower than observed upon incubation with the same concentration of 1′-OH estragole. HepG2 cells exposed to estragole E-3′-*N*^2^-dG was below the limit of detection (Fig. 3a).

### DNA adduct formation in induced liver cell models

**Fig. 4:**
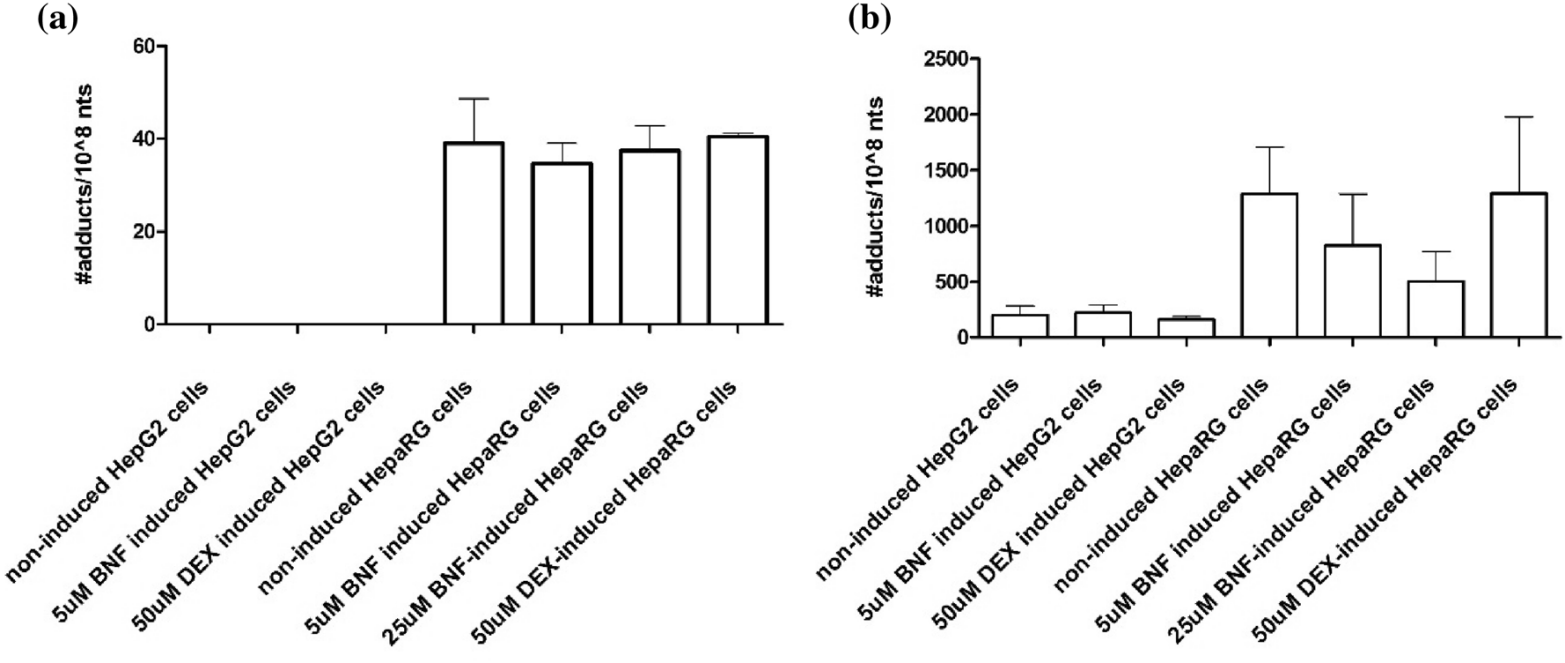
E-3′-*N*^2^-dG formation in HepG2 cells and HepaRG cells exposed to **a** 50 μM estragole or **b** 50 μM 1′-OH estragole for 2 h upon pretreatment with the CYP1A2 inducer BNF (final concentration 5 μM or 25 μM) or the CYP2A6 inducer DEX (final concentration 50 μM). Data represent the mean (+ SD) from three independent experiments. Note the 50-fold different scale of the Y-axis in figure **a** and **b**

Given that HepG2 and HepaRG models are easier to handle than rat hepatocytes, it was investigated whether E-3′-*N*^2^-dG formation in these models could be increased to the level observed in the primary rat hepatocytes upon induction of CYP2A6 or CYP1A2, known to convert estragole to 1′-OH estragole (Jeurissen et al. 2007). Thus, E-3′-*N*^2^-dG formation was studied in HepG2 cells and HepaRG cells pretreated with DEX or BNF in order to induce CYP2A6 or CYP1A2, respectively. Figure 4 presents the E-3′-*N*^2^-dG formation in induced HepG2 cells and induced HepaRG cells exposed to 50 μM estragole and 1′-OH estragole for 2 h. After 2 h of incubation with the parent compound estragole, E-3′-*N*^2^-dG formation was detected in HepaRG cells, but not in HepG2 cells, and DNA adduct formation in HepaRG cells was similar upon treatment with DEX or BNF as compared to non-induced control cells (Fig. 4a). In HepG2 cells, the DNA adduct levels formed upon 1′-OH estragole exposure were similar among the different treatments and also similar to the non-induced control. It is of interest to note that the results obtained upon exposure of the HepaRG cells to 1′-OH estragole revealed that pretreatment with BNF resulted in a reduction of the E-3′-*N*^2^-dG formation while DEX treatment had no effect (Fig. 4b). Comparison of the DNA adduct levels formed upon exposure of the HepaRG cells to estragole or 1′-OH estragole revealed that E-3′-*N*^2^-dG formation upon exposure to 1′-OH-estragole was again 15- to 37-fold higher than upon exposure to estragole. Based on these results it was concluded that the best cell models to study the formation and subsequent repair of E-3′-*N*^2^-dG adducts would be non-induced HepaRG cells and rat hepatocytes exposed to estragole.

### DNA repair

**Fig. 5:**
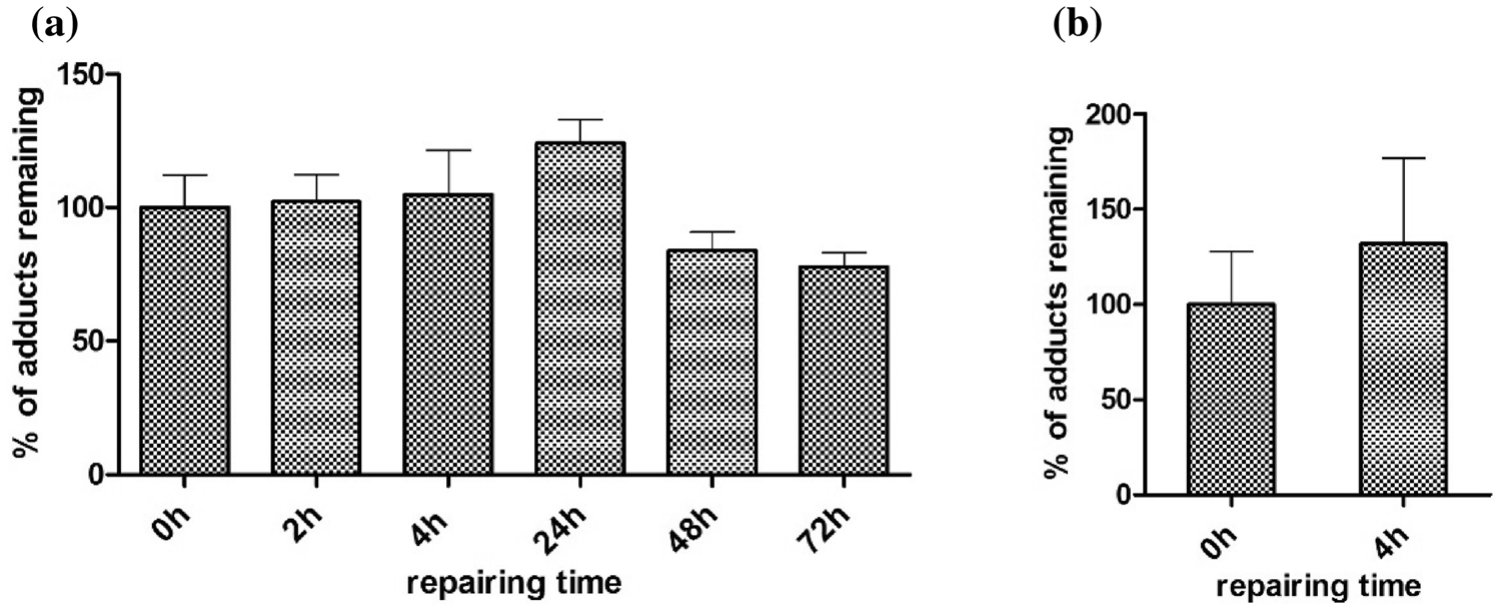
Time-dependent E-3′-*N*^2^-dG DNA adduct repair in **a** HepaRG cells and **b** primary rat hepatocytes. Raw data are available in supplementary information Table S1. Repair is expressed as the level of E-3′-*N*^2^-dG adducts remaining expressed as the percentage of the DNA adduct levels detected at 0 h, set at 100%. The level of DNA adducts at 0 h was 38 ± 5/10^8^ nts and 102 ± 28/10^8^ nts in HepaRG cells and rat hepatocytes, respectively. Data represent the average and SD from three independent experiments

Figure 5 shows the time-dependent change in DNA adduct levels in both HepaRG and rat hepatocytes after removal of estragole from the culture medium. In HepaRG cells (Fig. 5a), the level of E-3′-*N*^2^-dG gradually declined during the subsequent 72 h although the reduction did not reach statistical significance. After 72 h the repair was not complete and there was a substantial level of E-3′-*N*^2^-dG (77% of the original amount) remaining. In rat hepatocytes which can be kept in suspension for a limited amount of time only, DNA adduct levels upon 4 h repair were not reduced compared to the amount detected immediately upon the removal of estragole (Fig. 5b). The limited reduction in E-3′-*N*^2^-dG adducts upon periods of repair may be in part due to the NER mechanism, but could also be due to apoptosis removing cells with high levels of adducts from the population. These results indicate that in these liver cell models over the time span of the repair periods the E-3′-*N*^2^-dG adducts were repaired to only a limited extent, if at all.

### Role of NER in the repair of E-3′-*N*^2^-dG DNA adducts

In order to investigate the possible role of NER in the repair of E-3′-*N*^2^-dG DNA adducts the repair of E-3′-*N*^2^-dG DNA adducts was studied in CHO wild-type cells, and three NER-deficient mutants. Given that these cells do not contain substantial levels of cytochromes P450, E-3′-*N*^2^-dG levels were induced by incubating the cells with 1′-OH estragole. The concentration of 1′-OH estragole was chosen such that levels of the E-3′-*N*^2^-dG DNA adducts formed were comparable to what was detected in the HepaRG cells exposed to estragole. At 50 μM 1′-OH estragole the E-3′-*N*^2^-dG DNA adduct levels at 0 h amounted to 46 ± 6/10^8^ nts in wild-type cells, 34 ± 6/10^8^ nts in UV 5 cells, 30 ± 3/10^8^ nts in UV 24 cells and 32 ± 4/10^8^ nts in UV 41 cells.

**Fig. 6:**
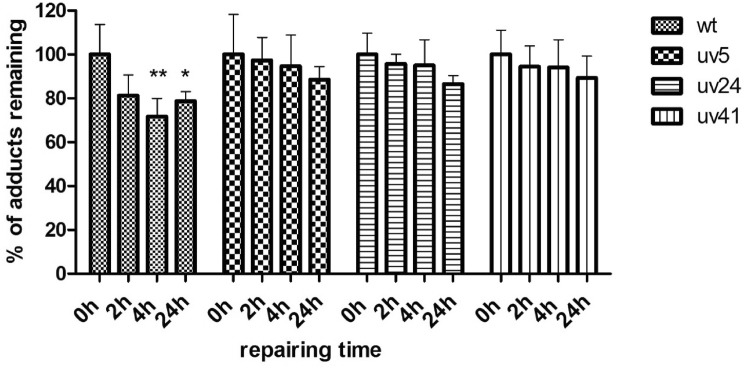
Time-dependent E-3′-*N*^2^-dG DNA adduct repair in CHO wild-type and NER-defective UV cells (UV 5, UV 24, and UV 41). Raw data are available in supplementary information Table S2. Repair is expressed as the level of E-3′-*N*^2^-dG adducts remaining expressed as the percentage of the DNA adduct levels detected at 0 h, set at 100%. Durnnett’s Multiple Comparison Test was used to test for significant difference in the DNA adduct levels between the 0 h repair sample and the samples with a certain number of hours repair for each cell line (*indicates *P* < 0.05); The level of DNA adducts at 0 h amounted to 46 ± 6/10^8^ nts in wild-type cells, 34 ± 6/10^8^ nts in UV 5, 30 ± 3/10^8^ nts in UV 24 and 32 ± 4/10^8^ nts in UV 41 based on four independent experiments

The results in Fig. 6 reveal that in CHO wild-type cells, there was a significant, albeit again limited, reduction in E-3′-*N*^2^-dG levels in the first 4 h after removal of the 1′-OH-estragole, with no further decrease up to 24 h recovery at which time around 80% of the DNA adducts (36 ± 2/10^8^ nts) remained in the cells. In corresponding NER-deficient cells, no significant NER repair was observed (Fig. 6). This indicates that NER might be involved in the E-3′-*N*^2^-dG adduct repair but that this process is eliminating the adducts over a 24-h time span to a limited extent only.

### Molecular modeling and MD simulations

To obtain further insight into the apparent inefficient repair of E-3′-*N*^2^-dG adducts, molecular modelling, and MD simulations were performed.

#### DNA-adduct structure

**Fig. 7:**
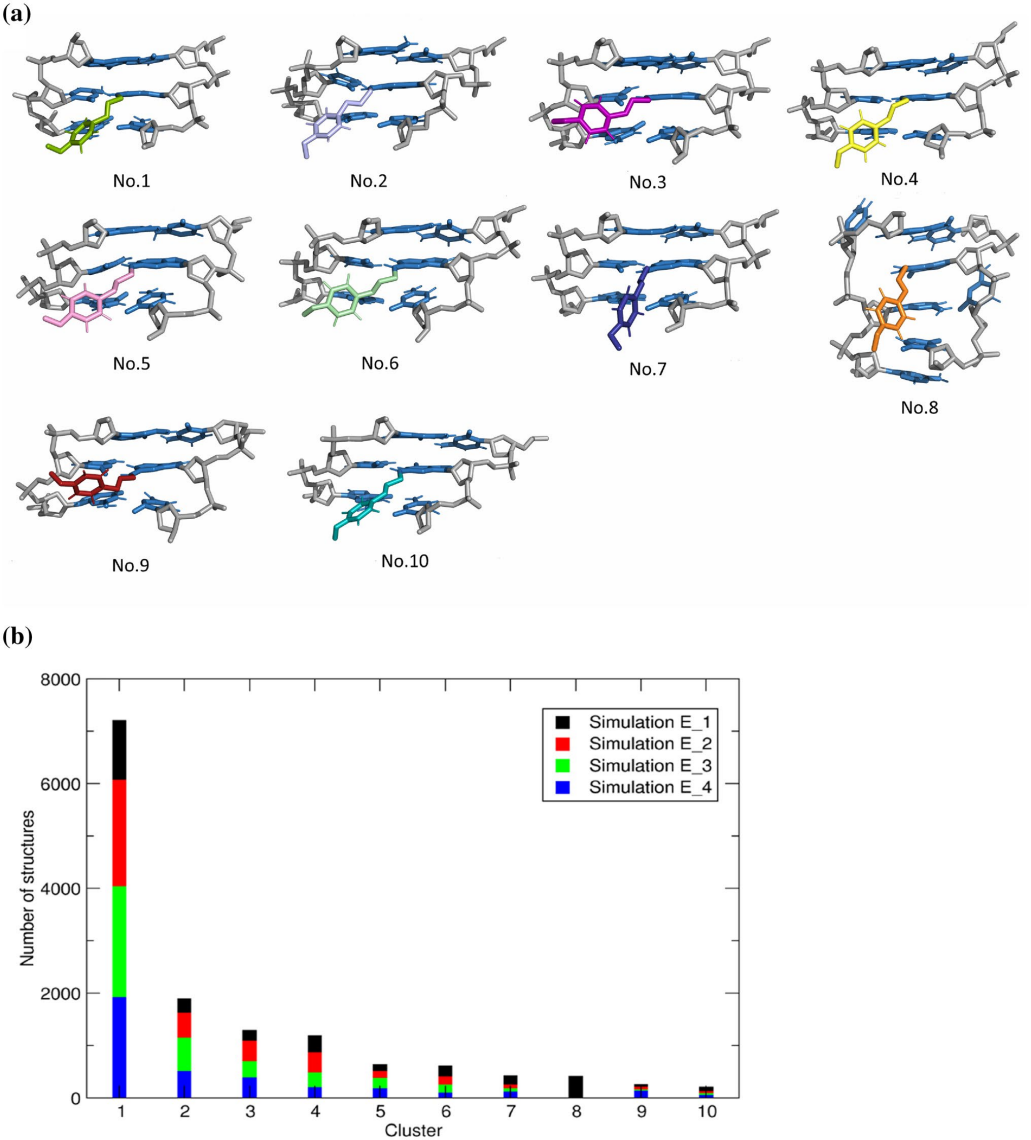
Ten representative E-3′-*N*^2^-dG adduct structures obtained from conformational clustering showing the central 3-mer (C5-G6*-C7) or the central 4-mer (C5-G6*-C7-T8) from the minor groove side (**a**), and the number of different conformations observed in the first ten clusters (**b**). Colors indicate the number of structures originating from the individual simulations (color figure online)

A conformational clustering was performed on the conformations of the E-3′-*N*^2^-dG adduct that were observed in each of the four simulations, starting from initial conformations E_1, E_2, E_3, and E_4. A total of 92 clusters were observed, of which the first ten clusters contained 88% of the sampled conformations. Figure 7a shows the representative structures of these ten clusters. The E-3′-*N*^2^-dG adduct incorporated into the selected base sequence resulted in the representative DNA adduct structures that shared similar binding features with the adduct residue remaining in the minor groove of the helix, aligned in 3′-direction. No intercalation of the adduct between the nucleobases was observed. Only one of the ten structures (NO. 8) showed base pair displacements with bases C7 and C17 flipped out into the major groove of the helix, respectively, and with the T8 base sandwiched between C7 and A15 (Fig. 7a). No base extrusion was found in the other structures. The number of structures in each cluster are shown in Fig. 7b. With 413 occurrences, cluster 8 represents 2.5% of all 16,000 analyzed conformations. It is interesting to note that except for cluster 8, all the clusters contained structures that originate from all four simulations, as indicated by the different colors. These results indicate that all the representative structures are sampled by the four simulations and that the simulations are not dependent on the initial structures. For cluster 8, 99.5% of the structures come from the simulations that started from the E_1 conformation. Apparently, a distortion of the DNA structure occurred in this simulation. The initial and final conformations of the individual simulations are shown in supplementary information Figs. S2 and S3. Supplementary information Table S3 presents the initial values of the dihedral angles *α*, *β*, and *δ*, and the interaction energy in the starting conformations E_1 to E_4. Table S3 presents the average values for each dihedral angle and the interaction energy along the whole simulation. The population distributions of the dihedral angles are shown in Supplementary information, Fig. S4. In agreement with the observation that all major clusters were sampled by each of the four simulations, the population distribution of the dihedral angle *α*, *β*, and *δ* are very similar for all simulations, with the possible exception of the simulation starting from conformation E_1.

#### RMSD analysis

**Fig. 8:**
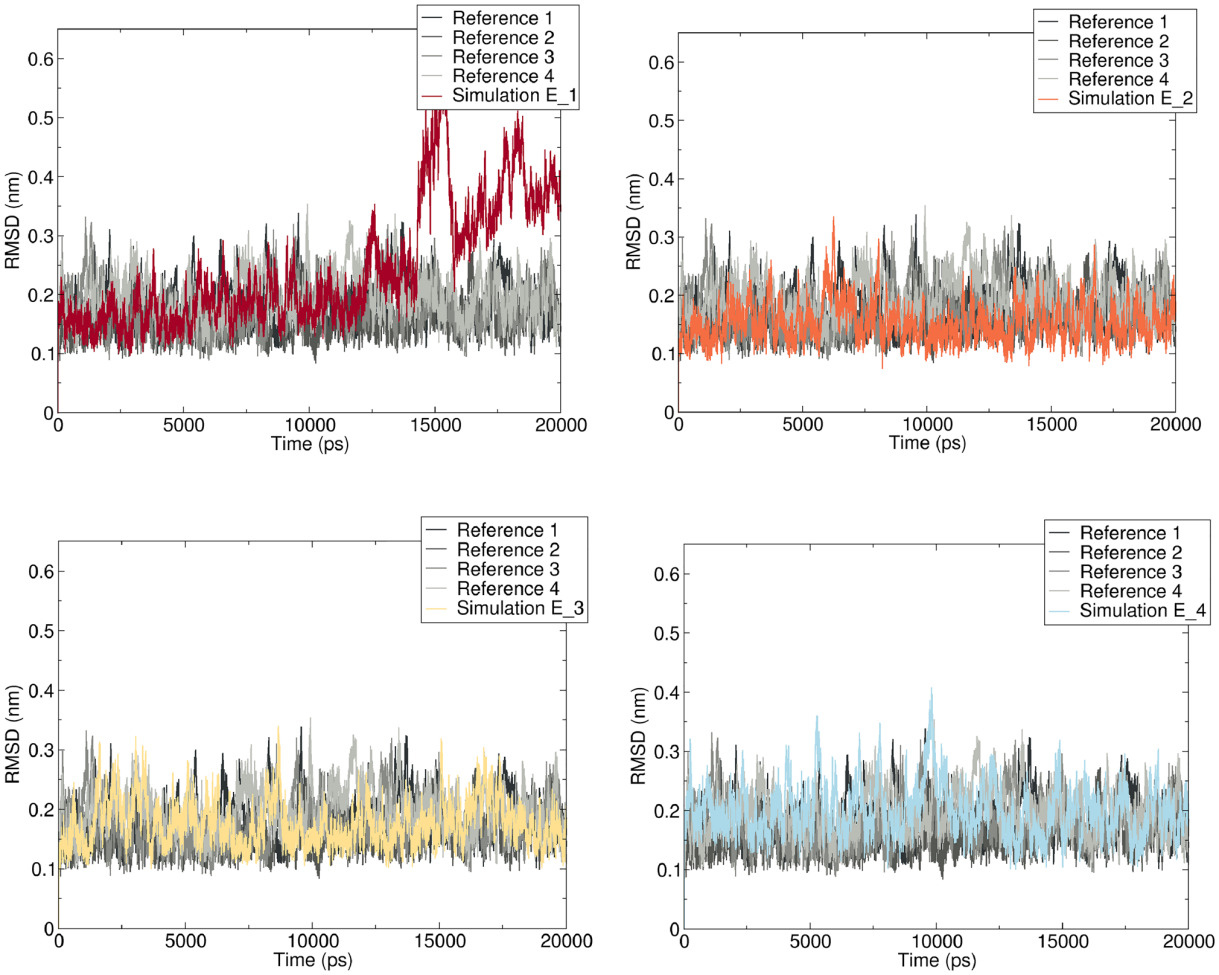
Time-dependent RMSD for four simulations of modified DNA duplex starting from different conformations of the E-3′-*N*^2^-dG adduct (colors) as well as the unmodified control duplex (grey scales) along the 20-ns simulation resulting in the final conformations depicted in Fig. S3 (color figure online)

An RMSD analysis was performed in order to quantify the structural differences between the DNA adduct conformations and reference DNA conformations. Time-dependent RMSD patterns for the different E-3′-*N*^2^-dG conformations, as well as for four replicates of the unmodified reference duplex were calculated along the MD trajectories for all the base pairs excluding the first and last one. Results obtained are presented in Fig. 8. For the reference duplex, the RMSD overall converged with values fluctuating around 0.15 nm. The relatively small increase of fluctuation in RMSD of the simulation E_1 occurred first at about 12 ns, which reflected the perturbation at T8:A15. The obvious fluctuation of the RMSD in the E_1 simulation occurred from 14 ns onwards until the end of the simulation and reflected the gradual displacement of the base pairs resulting in the final displacement (Supplementary information, Fig. S3 and movie 1). On the contrary, the RMSD for the other simulations starting from E_2 to E_4 revealed no differences with the reference duplex with overall RMSD values amounting to 0.15–0.2 nm on average.

#### Non-bonded interaction energy

**Fig. 9:**
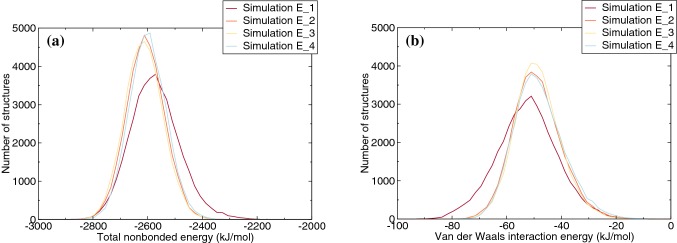
Population distribution of the non-bonded interaction energy (including Van der Waals interactions and electrostatic interactions) for the overall DNA helix excluding counter ions and solvent (**a**). Population distribution of total van der Waals interaction energy between estragole adduct residue and its surroundings (including ions) without solvent (**b**)

Total non-bonded interaction energy, including Van der Waals interactions and electrostatic interactions, of the DNA helix, as well as the Van der Waals interaction between adduct residue and its surroundings (excluding solvent) are shown in Fig. 9. Except for the simulation starting from conformation E_1, the other simulations presented similar energies in both analyses. It is interesting to notice that the simulation starting from the E_1 conformation shows more favorable Van der Waals interaction energy (Fig. 9b) but an overall less favorable non-bonded interaction energy for the entire structure (Fig. 9a). This indicates that although higher Van der Waals interaction energy may contribute to the stabilization of the DNA structure to some extent, the overall stability of DNA structure was impaired to a much larger extent. In Fig. S5, the population distributions for this simulation are split up into the initial (part I: 0–14 ns) and final (part II: 14–20 ns) parts, because according to Fig. 8, this is where the distortion of the structure appears. It is clear that the shifts that are seen in Fig. 9 originate from this distortion.

#### Hydrogen bonding

Table 1 shows the average occupancies of canonical (Watson–Crick) hydrogen bonds through the whole simulations in the middle 4-mer base pairs in both the modified and unmodified DNA duplex. The data obtained from the simulation E_1 reflect the distortion in hydrogen bonding from G6:C17 to T8: A15 with occupancies amounting to 38–80%. The most striking perturbation was found at T8:A15 rather than at the lesion site G6:C17, mainly resulting from the displacement of the base pair after approximately 12 ns, resulting in decreased average occupancies. Other simulations displayed only limited changes in the hydrogen bonding characteristics for all sites analyzed with the overall hydrogen bond occupancies within one base pair being similar in reference DNA and in modified DNA. In these conformations the decreases in hydrogen bond occupancies at N_4_–O_6_ were balanced by the increase at N_2_–O_2_. Overall the results indicate that upon E-3′-*N*^2^-dG adduct formation the hydrogen bonding was somewhat disturbed but only to a limited extent.

**Table 1:** Hydrogen bond occupancies (%) for the central 4-mer of the duplex DNA in different initial conformation simulations

		Ref	SD	Simulation E_1	Simulation E_2	Simulation E_3	Simulation E_4
C5…G18	N4..H41..O6	93.1	1.7	93.1	94.0	93.9	91.9
	N1..H1..N3	97.0	0.5	***95.0***	96.4	96.6	***96.0***
	N2..H21..O2	72.4	1.8	***62.5***	***70.6***	72.0	72.4
G6…C17	N4..H41..O6	92.6	0.7	***64.9***	***83.6***	***85.8***	***84.5***
	N1..H1..N3	98.7	0.1	***76.0***	***94.6***	***95.5***	***95.0***
	N2..H21..O2	88.2	0.7	***80.0***	*98.4*	*97.9*	*98.*2
C7…G16	N4..H42..O6	93.9	0.4	***65.5***	***91.3***	***91.8***	***91.9***
	N1..H1..N3	95.6	0.4	***71.8***	*96.9*	*97.*2	*96.7*
	N2..H21..O2	75.9	0.7	***61.3***	*80.8*	*80.7*	*79.3*
T8…A15	N6..H61..O4	92.5	0.6	***38.5***	*94.6*	93.2	93.4
	N3..H3..N1	96.3	0.2	***38.6***	***94.3***	***94.9***	***94.9***

Italic values represent values *higher* than the average of Ref plus standard deviation (SD; calculated over the four replicas of the simulations). Bold italic values represent values *lower* than the average of Ref minus SD. The hydrogen bonds for all the base pairs are shown in Supplementary information, Table S4

#### Structure classification

To further investigate on the structure classification changes in modified DNA as compared to reference DNA, the DISICL (DIhedral-based Segment Identification and Classification) algorithm was used to analyze the conformations. The two strands of the DNA duplex were classified separately (Table 2). In the first strand of the DNA duplex, the simulation starting from the E_1 conformation showed obvious changes with respect to the reference DNA simulations, where the sharp turn (ST) type gave a 3.5% increase at the expense of the AB transition class. The ST type defines segments where the backbone turns more than 90° (Nagy and Oostenbrink 2014), which is consistent with the observation of the DNA backbone connecting C17 and G18 bases, accompanied by the base extrusion (Fig. 7). A slight rise of the BL class (B-Loop) occupancy was observed in both simulations starting from the E_1 and E_3 conformations. Although the B-Loop cannot form the perfect B-helix, the increased percentage of B-Loop induces the structure shifting to become more like B-type helix (Nagy and Oostenbrink 2014). In the second strand, values of the unclassified (UC) and ST fractions in the simulation starting from the E_1 conformation increased with 5.2% and 0.9%, respectively, compared to the unmodified DNA, followed by a slight increase of the UC class (2.6%) in the E_2 simulation. Considering the two strands together, except for the E_1 simulation, there were no big changes in the structure classification of the other simulations.

**Table 2:** DNA structure classification of E-3′-*N*^2^-dG adduct and reference DNA conformations

Strand 1^a^	BI	BL	AH	ST	AB	UC
Ref	21.7 (7.1)	17.9 (1.7)	8.3 (5.5)	0.6 (0.72)	40.1 (6.5)	4.2 (1.3)
Simulation E_1	26.4	2*1.3*	3.8	*4.1*	***29.3***	5.4
Simulation E_2	28.2	18.0	5.8	0.5	38.2	3.7
Simulation E_3	20.5	2*1.4*	7.3	0.5	35.9	5.1
Simulation E_4	22.9	18.8	7.5	0.4	35.9	4.4

Strand 2	BI	BL	AH	ST	AB	UC
Ref	20.7 (11.9)	12.6 (3.7)	11.7 (8.3)	0.4 (0.3)	43.5 (8.38)	3.9 (2.0)
Simulation E_1	18.8	14.4	9.9	*1.5*	37.4	*9.1*
Simulation E_2	23.3	16.1	8.9	0.4	38.6	*6.5*
Simulation E_3	22.0	10.3	11.4	0.3	46.8	3.3
Simulation E_4	20.2	13.8	10.6	0.3	43.1	4.1

The percentage of six structure classifications is shown in the table. The complete classification is given in Table S5, Supplementary information. Data are presented as the average (standard deviation; SD) based on the middle nine residues. For the reference DNA, four replicates were performed. Values, higher than the average of Ref plus SD, shown in italic; lower than the average of Ref minus SD, shown in bolditalic

*BI* classical B form DNA, *BL* B-loop, *AH* A-helix, *ST* sharpturn, *AB* AB transition, *UC* unclassified

^a^Strand 1 contains the DNA adduct

#### Helicoidal parameters and groove width

**Fig. 10:**
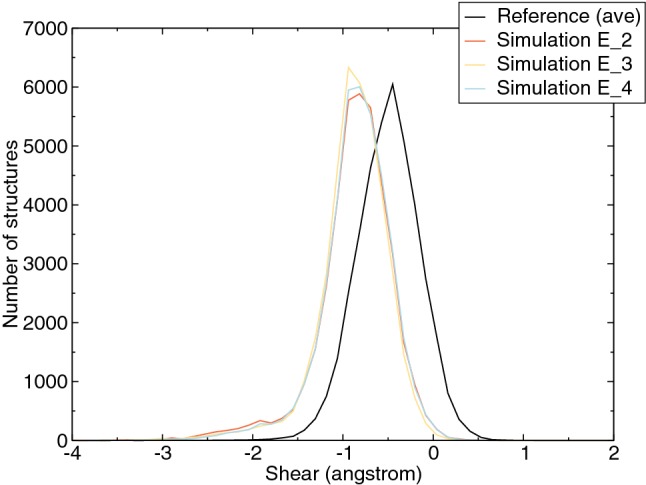
Distribution of observed Shear distances (in Å) at the G6:C17 base pair for the reference DNA simulations and the simulations starting from conformations E_2, E_3, and E_4

Helicoidal parameters, including six local base pair parameters (shear, stretch, stagger, buckle, propeller, and opening) and six step parameters (rise, roll, shift, slide, tilt, and twist) (Lu and Olson 2003), were measured at the center 3-mer for each conformation of modified DNA, reference DNA and for a classical B-type DNA. The definition of base pair step is shown in Fig. S6. The simulation starting from conformation E_1 was not included since the lack of base pairing information resulting from the base extrusion cannot provide the base-pair geometric position, consequently X3DNA cannot characterize the helicoidal parameters and groove width (Lu and Olson 2003). Among the local base pair parameters, an obvious enlargement of the shear distance compared to the reference DNA only occurred at the lesion site in all the simulations (Fig. 10). Shear is one of the critical parameters characterizing the hydrogen bonding features (Lu and Olson 2003), which is in agreement with the observed loss of hydrogen bonds at the G6:C17 base pair in all simulations (Table 1). Except shear, small differences were also observed in buckle (all the center base pairs) and stagger (G6 and C7 base pairs), see Fig. S7. For the step parameters small differences were seen in rise, shift (C5–G6 step), slide, and tilt (G6–C7 step). Interestingly, these parameters showed the structure of modified DNA were slightly more like the classical B-type form DNA (Supplementary information, Table S6).

Groove widths were measured as the smallest phosphorus–phosphorus separation between the two strands through minor and major groove. Both major and minor groove widths were comparable with the reference DNA simulations in all modified DNA simulations. The simulation starting from conformation E_1 is excluded based on the same reason as presented for the helicoidal parameters measurement (Supplementary information, Fig. S8).

## Discussion

Insight into the formation and repair of estragole DNA adducts is essential to better understand the consequences of daily dietary exposure to this food-borne genotoxic carcinogen present in herbs and spices and products derived from them. Earlier in vitro studies on DNA adduct formation by alkenylbenzenes were performed with primary rat hepatocytes or HepG2 cells (Alhusainy et al. 2013; Cartus et al. 2012; Jeurissen et al. 2008; Paini et al. 2010). In the present study, on estragole DNA adduct formation and repair also HepaRG cells were included since these cells can differentiate into cells with hepatocyte-like morphology when treated with DMSO and are reported to express cytochrome P450 and phase II enzymes at levels more comparable to liver hepatocytes than HepG2 cells (Kanebratt and Andersson 2008; Nelson et al. 2017). Data obtained revealed that formation of E-3′-*N*^2^-dG DNA adducts is increasing in the order HepG2 cells < HepaRG cells < primary rat hepatocytes upon exposure to either estragole or 1′-OH estragole. In all cell models, DNA adduct formation was readily detectable upon exposure to 1′-OH estragole, indicating the cells contain the SULT activity required for conversion of 1′-OH estragole to the DNA reactive 1′-sulfoxy metabolite. In HepaRG cells and primary rat hepatocytes, DNA adduct formation was also readily observed upon estragole exposure with levels formed in hepatocytes being only 2.5-fold higher than those in the HepaRG cells. The lack of formation of detectable E-3′-*N*^2^-dG levels in HepG2 cells likely reflects the relatively low CYP activity in these cells (Westerink and Schoonen 2007) hampering the efficient conversion of the parent compound to the 1′-OH-metabolite.

Pretreatment of the HepG2 or HepaRG cells with BNF and DEX to induce CYP1A2 and CYP2A6 (Gerets et al. 2012; Meunier et al. 2000), the enzymes shown previously to be involved in estragole 1′-hydroxylation (Jeurissen et al. 2007) did not result in increased levels of E-3′-*N*^2^-dG formation. Pretreatment of HepaRG cells with BNF even resulted in a decrease in the E-3′-*N*^2^-dG formation upon exposure to 1′-OH estragole, likely resulting from induction of uridine diphosphate-glucuronosyl transferase (UDPGT) or glutathione S-transferases (GST) which is able to support detoxification pathways (Takahashi et al. 1996), but this was not further investigated. Based on the induction results, the non-induced HepaRG cells and rat hepatocytes were used in the DNA repair assays instead of further developing or investigating the actual level of the respective CYPs in the induced cell models.

Based on these results, subsequent studies on repair of E-3′-*N*^2^-dG adducts were performed in HepaRG and primary hepatocytes exposed to estragole. Repair of the alkenylbenzene DNA adducts is expected to proceed via nucleotide excision repair (NER), and the role of NER in the repair of E-3′-*N*^2^-dG DNA adducts was investigated using NER-proficient CHO wild-type and NER-deficient CHO UV cell lines. The results obtained with these cell models reveal that NER contributes to repair of the E-3′-*N*^2^-dG DNA adducts, which might be induced especially during cell check point (Barnum and O’Connell 2014), but that it was only reducing the E-3′-*N*^2^-dG DNA adduct levels in the CHO wild-type cells to about 70–80% of the original adduct levels, in the 24 h following their formation. The same result was obtained for the HepaRG cells exposed to estragole, in which upon 48- and 72-h repair 84 and 78% of the E-3′-*N*^2^-dG DNA adducts persisted. Also in primary hepatocytes, the DNA adduct level was not reduced after 4 h repair. This observation in the in vitro cell models is in line with in vivo data reporting DNA adducts in the liver of CD-1 mice exposed to the related alkenylbenzene safrole to be persisted up to 30 days (Gupta et al. 1993). For estragole, Phillips et al. (1981) reported that *N*^2^-guanine adducts, including E-3′-*N*^2^-dG adducts, formed in the liver of mice exposed intravenously to 1′-OH estragole, can be removed, although a significant fraction of the adducts persisted up to at least 20 days after treatment. These authors already suggested that the relatively inefficient repair of the E-3′-*N*^2^-dG DNA adducts could be the consequence of the conformation of the DNA adducts (Phillips et al. 1981). Also for other DNA adducts, including for example 10S-(–)-*trans*-B[a]PDE-*N*^2^-dG, it has been suggested that efficient binding of XPC-Hhr23B at the site of the DNA lesion is hampered when the DNA adducts formed do not result in substantial base displacement (Min and Pavletich 2007; Mu et al. 2015).

To further investigate the possible conformation dependent (in)efficiency of E-3′-*N*^2^-dG DNA adduct repair, molecular modelling and MD simulation were applied to characterize the structural changes resulting from formation of these adducts in the double-stranded DNA helix.

The results from molecular simulations indicated that the E-3′-*N*^2^-dG DNA adduct is likely to insert sideways in the DNA duplex and protrudes out of the minor groove with the estragole moiety orienting towards to 3′-side direction. Most of the representative structures seen in the first ten clusters correspond to stable DNA double helices. One simulation (starting from conformation E_1) displays obvious structure distortion emerging during the last 6 ns of the 20-ns simulation. This distortion is accompanied by displacement of T8:A15 with T8 intercalating between C7 and A15 bases. At the same time, part of the phenyl ring of the estragole moiety gets closer to the base opposite the modified site, resulting in disturbance of the G6*:C17 base pair, and extrusion of C17 base out of the helix towards the minor groove. The other part of the phenyl ring of estragole gets closer to the C7 base, causing extrusion of the C7 to the major groove (Supplementary information, movie 2). This whole process is correlated with severe hydrogen bond disruption and with a greater extent of unwinding.

It is interesting to notice that NER can recognize different lesions without the need for a common chemical motif, because the critical factor for recognition, XPC-Hhr23B, is triggered by destabilization of the DNA helix instead of by the damage itself (Nasheuer 2009). The term *destabilization* emphasizes the structural distortion of the secondary structure of the DNA, mainly caused by loss of the Watson–Crick hydrogen bonding and the local instability induced by the lesions (Mocquet et al. 2007). In the present study, the RMSD, structure classification and non-bonded interaction energy reflecting the overall stability of the DNA structure, all point at a severe distortion occurring in only one out of four simulations. At the local lesion site, this simulation shows flipped bases on both the damaged and undamaged strands. Similar to the ( +)-*cis*-B[a]PDE-*N*^2^-dG adduct, a good NER substrate, it results in disruption of the Watson–Crick base pair, and extrusion of the flipped partner base of the modified guanine into the major groove (Mu et al. 2017). This flipped base on the undamaged strand is a key conformational property for XPC-Hhr23B binding and recruits later NER factors including TFIIH, XPA and XPG to the lesion site (Buterin et al. 2005). Therefore, the character of bases extrusion in conformations obtained from simulation E_1 could assist XPC- Hhr23B protein capture in an efficient way and induce NER response. On the contrary, for (–)-*trans*-B[a]PDE-*N*^2^-dG, which is repaired by NER with five to ten times lower efficiency than ( +)-*cis*-B[a]PDE-*N*^2^-dG (Hess et al. 1997; Mocquet et al. 2007), a structural conformation was obtained in which the adduct residue is positioned in the minor groove, aligned in 3′-direction without base displacement, with the Watson–Crick hydrogen bonding of all base pairs remaining intact (Andreas 2005; Mocquet et al. 2007). These observations are very similar to the structures observed in nine of the first ten clusters in Fig. 7 for the E-3′-*N*^2^-dG DNA adducts. For these conformations, no base extrusion appeared and all Watson–Crick hydrogen bonds remained in place. Data from the central 3-mer helicoidal parameters reveal that the adduct insertion even impacts the DNA structure to more closely resemble the canonical B-type DNA. Therefore, the NER responses towards these conformations are likely weaker than towards the conformations observed in the simulation starting from conformation E_1. Together, our MD simulation data do show that the distortion of the DNA structure is limited because only in the simulation starting from conformation E_1 a distortion was observed for 8 ns of the 20 ns. Given that distortion of the DNA double helix is the trigger for NER mediated repair, the limited distortion of the DNA helix in terms of both conformational changes and time during which they were observed in the MD simulations, provides a qualitative explanation for the limited DNA repair. In line with what was previously shown for other NER resistant DNA adducts (Mu et al. 2017; Mocquet et al. 2007) the current MD simulations provide an initial analysis of the adducted state in terms of conformational changes, hydrogen-bond disruptions, non-bonded interaction energy and structure classification at a 20-ns simulation time scale. Our observations support a qualitative link between the limited NER mediated repair and the DNA distortion by E-3′-*N*^2^-dG adduct formation. Linking the outcomes of the MD simulation in a quantitative way to the experimental data is not possible based on the current simulation data. First of all, the relative occurrence of the different conformations of the E-3′-*N*^2^-dG DNA adduct or quantification of which one is the dominant one is not revealed in this study and would require a significantly increased computational effort. Significantly more and longer simulations would be required. Second, it is not known exactly what extent of distortion is needed to activate NER mediated repair, further hampering a quantitative comparison. Moreover, other factors than limited NER mediated repair, like apoptosis and/or DNA/cell replication, may have an influence on the adduct level, again hampering a quantitative comparison.

Except conformational aspects, other factors may also impact the potential efficiency of NER repair, including the base sequence context where the lesion is located (Cai et al. 2010; Liu et al. 2015; Ruan et al. 2007) and steric crowding that occurs if more than one guanine is modified in the same strand (Kropachev et al. 2009). Since it can be expected that adduct formation is a stochastic process it can also be foreseen that E-3′-*N*^2^-dG adducts in reality will be present within a large variety of DNA backgrounds. Thus the results of the present study obtained using the 11 mer provides a first indication on structural conformational changes to be expected upon E-3′-*N*^2^-dG adduct formation. Studying the consequences of variability in this 11-mer for the structural changes upon E-3′-*N*^2^-dG formation, and the resulting chances on DNA repair, remains an interesting topic for future research. Using the now defined 11-mer provides the advantage of enabling comparison of the outcomes to similar studies performed previously for benzo[a]pyrene DNA adducts (Mocquet et al. 2007; Mu et al. 2017). The local thermodynamic stability of the DNA helix (Cai et al. 2012; Reeves et al. 2011) and steps in the NER pathway following recognitions of the DNA damage are possible factors that can be involved in the NER repair efficiency in general.

Finally, it is of importance to note that inefficient repair of the DNA damage caused by estragole is likely to contribute to the ultimate hazards and risks of this food-borne dietary ingredient. It implies that repair will not be complete before a subsequent dietary exposure, or before cell division occurs, providing possibilities for accumulation of the damage and increasing the chances on the DNA damage turning into a mutation.

## Electronic supplementary material

Below is the link to the electronic supplementary material.

Supplementary file1 (DOCX 14606 kb)

Supplementary file2 (MPG 3370 kb)

Supplementary file3 (MP4 15940 kb)
