# Supplementary material for: Cellular levels and molecular dynamics simulations of estragole DNA adducts point at inefficient repair resulting from limited distortion of the double-stranded DNA helix
Source: Arch Toxicol. 2020 Mar 18;94(4):1349–65. doi: 10.1007/s00204-020-02695-5 (PMC7225201; doi:10.1007/s00204-020-02695-5)
Supplement: Supplementary file 1 — Supplementary file1 (DOCX 14606 kb) [file 204_2020_2695_MOESM1_ESM.docx]

**Supplementary information to**

Cellular levels and molecular dynamics simulations of estragole DNA adducts point at inefficient repair resulting from limited distortion of the double strand DNA helix

Shuo Yang,^1^ Matthias Diem,^2^ Jakob D.H. Liu,^2^ Sebastiaan Wesseling,^1^ Jacques Vervoort,^3^ Chris Oostenbrink,^2^ Ivonne M.C.M. Rietjens^1^

1. Division of Toxicology, Wageningen University, Stippeneng 4, 6708 WE Wageningen, The Netherlands

2. Division of Material Sciences and Process Engineering, University of Natural Resources and Life Science, Vienna

3. Division of Biochemistry, Wageningen University, Stippeneng 4, 6708 WE Wageningen, The Netherlands

**Table S 1** Three time-dependent E-3’-N^2^-dG DNA adduct repair in HepaRG cells and primary rat hepatocytes

|  | HepaRG cells | | | | | |
| --- | --- | --- | --- | --- | --- | --- |
| Independence | 0h | 2h | 4h | 24h | 48h | 72h |
| 1^st^ | 41.1 | 38.6 | 43.0 | 51.9 | 35.6 | 27.9 |
| 2^nd^ | 41.7 | 43.9 | 33.2 | 45.4 | 31.0 | 32.0 |
| 3^rd^ | 33.3 | 36.3 | 45.5 | 46.8 | 31.0 | 30.5 |
| Average | 38.7 | 39.6 | 40.6 | 48.0 | 32.5 | 30.1 |
| SD | 4.7 | 3.9 | 6.5 | 3.4 | 2.6 | 2.1 |

| Rat hepatocytes | | |
| --- | --- | --- |
| Independence | 0h | 4h |
| 1^st^ | 128.0 | 175.0 |
| 2^nd^ | 108.2 | 146.7 |
| 3^rd^ | 71.9 | 84.6 |
| Average | 102.7 | 135.4 |
| SD | 28.5 | 46.3 |

**Table S 2** Four time-dependent E-3’-N^2^-dG DNA adduct repair in CHO wild type and NER-defective UV cells (UV 5, UV 24 and UV 41)

| CHO wild type | | | | |
| --- | --- | --- | --- | --- |
| Independence | 0h | 2h | 4h | 24h |
| 1^st^ | 52.2 | 42.1 | 27.3 | 35.8 |
| 2^nd^ | 46.1 | 38.0 | 34.8 | 38.4 |
| 3^rd^ | 48.4 | 31.5 | 34.9 | 36.8 |
| 4^th^ | 37.3 | 37.8 | 34.9 | 33.6 |
| Average | 46.0 | 37.3 | 33.0 | 36.2 |
| SD | 6.3 | 4.3 | 3.8 | 2.0 |
| CHO UV5 | | | | |
| Independence | 0h | 2h | 4h | 24h |
| 1^st^ | 30.9 | 38.4 | 34.0 | 41.3 |
| 2^nd^ | 43.1 | 31.4 | 33.9 | 29.4 |
| 3^rd^ | 32.8 | 31.3 | 35.8 | 32.4 |
| 4^th^ | 29.2 | 31.3 | 25.1 | 28.6 |
| Average | 34.0 | 33.1 | 32.2 | 32.9 |
| SD | 6.2 | 3.5 | 4.8 | 5.8 |
| CHO UV41 | | | | |
| Independence | 0h | 2h | 4h | 24h |
| 1^st^ | 29.3 | 31.6 | 30.3 | 27.2 |
| 2^nd^ | 32.1 | 28.9 | 29.6 | 25.8 |
| 3^rd^ | 30.0 | 27.1 | 25.5 | 28.3 |
| 4^th^ | 37.2 | 32.9 | 35.5 | 33.3 |
| Average | 32.1 | 30.4 | 30.2 | 28.7 |
| SD | 3.6 | 3.0 | 4.1 | 3.2 |
| CHO UV24 | | | | |
| Independence | 0h | 2h | 4h | 24h |
| 1^st^ | 33.9 | 27.3 | 31.0 | 25.2 |
| 2^nd^ | 27.7 | 28.4 | 26.0 | 25.0 |
| 3^rd^ | 29.3 | 30.3 | 31.5 | 41.3 |
| 4^th^ | 28.6 | 29.1 | 26.1 | 29.2 |
| Average | 29.9 | 28.8 | 28.7 | 30.2 |
| SD | 2.8 | 1.2 | 3.0 | 7.6 |

**Table S 3** The initial and average dihedral angles with standard deviation over the simulation in brackets and total interaction energy of the adducted base for the different simulations including the initial and average values consisting of the complete non-bonded energy of the modified base G6* with its surroundings, as well as the covalent torsion energy term

| Simulations | Torsional angle (α)  [°] | Torsional angle (β)  [°] | Torsional angle (γ)  [°] | Torsional angle (δ)  [°] | Interaction energy [kJ/mol] |
| --- | --- | --- | --- | --- | --- |
| E_1 (initial) | 10 | 130 | 180 | 90 | -124.1 |
| E_1 (average) | 25.2 (27.9) | 33.1 (95.5) | 181.8 (8.5) | -13.3 (112.8) | -894.7 (42.5) |
| E_2 (initial) | 60 | -80 | -180 | 30 | -122.9 |
| E_2 (average) | 17.1 (22.8) | 21.7 (87.6) | 181.8 (8.9) | -14.3 (110.7) | -901.2 (41.2) |
| E_3 (initial) | 30 | -160 | 180 | 60 | -116.4 |
| E_3 (average) | 18.2 (22.7) | 14.4 (83.6) | 181.5 (9.2) | -19.7 (117.1) | -902.2 (40.8) |
| E_4 (initial) | 60 | -50 | 180 | 120 | -137.6 |
| E_4 (average) | 15.7 (25.7) | 19.5 (87.0) | 181.6 (8.9) | -16.0 (111.0) | -901.7 (41.2) |

**Table S 4** Hydrogen bond occupancies (%) for the duplex DNA in different initial conformation simulations. Blue highlighted values represent values lower than the average of Ref minus SD, yellow highlighted values represent values higher than the average of Ref plus SD

|  |  | Ref | SD | Simulation E_1 | Simulation E_2 | Simulation E_3 | Simulation E_4 |
| --- | --- | --- | --- | --- | --- | --- | --- |
| C1-G22 | N4..H41..O6 | 86.47 | 1.56 | 87.61 | 87.3 | 88.35 | 88.82 |
|  | N1..H1..N3 | 95.15 | 1.31 | 95.6 | 94.23 | 94.42 | 95.12 |
|  | N2..H21..O2 | 68.45 | 1.72 | 68.17 | 68.98 | 68.45 | 67.6 |
| C2-G21 | N4..H41..O6 | 89.23 | 3.18 | 79.19 | 91.73 | 93.8 | 88.24 |
|  | N1..H1..N3 | 94.35 | 2.34 | 84.86 | 97.14 | 97.66 | 93.85 |
|  | N2..H21..O2 | 76.05 | 2.37 | 72.19 | 80.14 | 78.23 | 76.33 |
| A3-T20 | N6..H61..O4 | 86.18 | 3.72 | 91.23 | 90.81 | 86.8 | 89.61 |
|  | N3..H3..N1 | 92.46 | 3.49 | 92.94 | 92.61 | 93.14 | 92.88 |
| T4-A19 | N6..H61..O4 | 94.12 | 1.22 | 94.95 | 94.62 | 93.38 | 94.68 |
|  | N3..H3..N1 | 93.10 | 0.60 | 93.37 | 92.51 | 93.05 | 92.49 |
| C5-G18 | N4..H41..O6 | 93.13 | 1.66 | 93.1 | 93.97 | 93.88 | 91.91 |
|  | N1..H1..N3 | 97.02 | 0.48 | 94.98 | 96.42 | 96.62 | 95.95 |
|  | N2..H21..O2 | 72.45 | 1.76 | 62.5 | 70.55 | 72 | 72.39 |
| G6-C17 | N4..H41..O6 | 92.61 | 0.70 | 64.93 | 83.56 | 85.76 | 84.46 |
|  | N1..H1..N3 | 98.74 | 0.14 | 75.95 | 94.59 | 95.48 | 95 |
|  | N2..H21..O2 | 88.16 | 0.68 | 79.72 | 98.36 | 97.87 | 98.17 |
| C7-G16 | N4..H42..O6 | 93.86 | 0.39 | 65.54 | 91.32 | 91.82 | 91.91 |
|  | N1..H1..N3 | 95.59 | 0.43 | 71.75 | 96.91 | 97.15 | 96.69 |
|  | N2..H21..O2 | 75.85 | 0.73 | 61.31 | 80.79 | 80.71 | 79.25 |
| T8-A15 | N6..H61..O4 | 93.53 | 0.57 | 38.53 | 94.59 | 93.24 | 93.4 |
|  | N3..H3..N1 | 96.28 | 0.20 | 38.57 | 94.3 | 94.9 | 94.93 |
| A9-T14 | N6..H61..O4 | 92.28 | 1.29 | 84.73 | 94.25 | 91.7 | 89.1 |
|  | N3..H3..N1 | 87.01 | 0.80 | 89.75 | 91.74 | 88.85 | 86.22 |
| C10-G13 | N4..H41..O6 | 88.83 | 0.73 | 84.54 | 87.96 | 88.83 | 88.35 |
|  | N1..H1..N3 | 95.24 | 0.38 | 95.72 | 95.56 | 95.63 | 95.85 |
|  | N2..H21..O2 | 73.60 | 1.20 | 77.19 | 76.93 | 73.99 | 75.91 |
| C11-G12 | N4..H41..O6 | 87.25 | 0.57 | 86.65 | 87.59 | 87.08 | 87.89 |
|  | N1..H1..N3 | 96.28 | 0.22 | 96.19 | 96.54 | 96.44 | 96.3 |
|  | N2..H21..O2 | 71.95 | 0.62 | 70.38 | 72.91 | 71.58 | 71.86 |

**Table S 5** Detailed DISICL Classes for reference DNA and modified DNA classification, and abbreviations (code). Data are presented as the average percentage of occupancies for the central 9-mer. Four independent simulations were performed in reference DNA, and each simulation with different starting conformations was performed once in modified DNA

First strand

| Class | Code | Ref (SD) | Simulation E_1 | Simulation E_2 | Simulation E_3 | Simulation E_4 |
| --- | --- | --- | --- | --- | --- | --- |
| BI-helix | BI | 21.73 (7.11) | 26.40 | 28.24 | 20.50 | 22.92 |
| BII-helix | BII | 2.52 (1.73) | 2.83 | 1.90 | 2.90 | 3.18 |
| BIII-helix | BIII | 0.68 (0.30) | 0.81 | 0.73 | 1.03 | 0.77 |
| B-loop | BL | 17.90 (5.16) | 21.27 | 18.01 | 21.40 | 18.77 |
| A-helix | AH | 8.25 (5.50) | 3.76 | 5.80 | 7.34 | 7.50 |
| A-loop | AL | 0.42 (0.14) | 0.72 | 0.40 | 0.42 | 0.39 |
| Z-helix | ZH | 0.00 (0.00) | 0.00 | 0.00 | 0.00 | 0.00 |
| quad loop | QL | 0.00 (0.00) | 0.80 | 0.00 | 0.00 | 0.00 |
| sharp turns | ST | 0.61 (0.72) | 4.07 | 0.53 | 0.46 | 0.42 |
| tetraloop B | TL | 0.10 (0.05) | 1.30 | 0.10 | 0.12 | 0.13 |
| AB trans | AB | 40.12 (6.53) | 29.31 | 38.17 | 35.91 | 35.87 |
| AB2 trans | AB2 | 3.18 (1.59) | 2.69 | 2.24 | 3.56 | 3.44 |
| AZ trans | AZ | 0.00 (0.00) | 0.07 | 0.00 | 0.00 | 0.00 |
| ZB trans | ZB | 0.00 (0.00) | 0.00 | 0.00 | 0.00 | 0.00 |
| AD trans | AD | 0.04 (0.06) | 0.12 | 0.00 | 0.24 | 0.34 |
| BD trans | BD | 0.25 (0.29) | 0.37 | 0.13 | 0.96 | 1.78 |
| ZD trans | ZD | 0.00 (0.00) | 0.00 | 0.00 | 0.00 | 0.00 |
| Unclassified | UC | 4.18 (1.29) | 5.37 | 3.72 | 5.08 | 4.42 |

Second strand

| Class | Code | Ref (SD) | Simulation E_1 | Simulation E_2 | Simulation E_3 | Simulation E_4 |
| --- | --- | --- | --- | --- | --- | --- |
| BI-helix | BI | 20.67 (11.89) | 18.81 | 23.27 | 22.00 | 20.24 |
| BII-helix | BII | 2.72 (3.67) | 2.62 | 1.72 | 1.53 | 2.84 |
| BIII-helix | BIII | 0.43 (0.29) | 0.47 | 0.82 | 0.26 | 0.56 |
| B-loop | BL | 12.63 (3.68) | 14.41 | 16.07 | 10.30 | 13.82 |
| A-helix | AH | 11.71 (8.34) | 9.89 | 8.88 | 11.44 | 10.57 |
| A-loop | AL | 0.49 (0.27) | 1.23 | 0.46 | 0.46 | 0.48 |
| Z-helix | ZH | 0.00 (0.00) | 0.00 | 0.00 | 0.00 | 0.00 |
| quad loop | QL | 0.00 (0.00) | 0.06 | 0.00 | 0.00 | 0.00 |
| sharp turns | ST | 0.36 (0.30) | 1.50 | 0.41 | 0.33 | 0.33 |
| tetraloop B | TL | 0.13 (0.08) | 0.40 | 0.12 | 0.09 | 0.12 |
| AB trans | AB | 43.48 (8.38) | 37.43 | 38.61 | 46.82 | 43.10 |
| AB2 trans | AB2 | 3.11 (2.09) | 3.80 | 2.91 | 2.59 | 3.40 |
| AZ trans | AZ | 0.00 (0.00) | 0.00 | 0.00 | 0.00 | 0.00 |
| ZB trans | ZB | 0.00 (0.00) | 0.01 | 0.00 | 0.00 | 0.00 |
| AD trans | AD | 0.01 (0.01) | 0.03 | 0.07 | 0.12 | 0.18 |
| BD trans | BD | 0.26 (0.39) | 0.24 | 0.20 | 0.77 | 0.23 |
| ZD trans | ZD | 0.00 (0.00) | 0.00 | 0.00 | 0.00 | 0.00 |
| Unclassified | UC | 3.89 (1.99) | 9.07 | 6.50 | 3.30 | 4.14 |

**Table S 6** Average values of duplex helicoidal parameters for reference DNA (with standard deviations), modified DNA and classical B-type DNA. Only values having a clear difference between reference DNA and modified DNA are shown in the table

| Buckle | Simulation E_2 | Simulation E_3 | Simulation E_4 | Reference DNA | Classical B-type DNA |
| --- | --- | --- | --- | --- | --- |
| C5:G18 | 11.38 | 13.84 | 11.87 | 15.35 (1.33) | -0.45 |
| G6:C17 | -6.54 | -8.86 | -8.25 | -10.85 (1.02) | 0.45 |
| C7:G16 | 3.41 | 3.24 | 2.87 | -2.03 (0.63) | -0.47 |
| Shear |  |  |  |  |  |
| G6:C17 | -0.93 | -0.94 | -0.92 | -0.56 (0.02) | -0.14 |
| Stagger |  |  |  |  |  |
| G6:C17 | -0.13 | -0.17 | -0.16 | -0.25 (0.02) | 0.09 |
| C7:G16 | 0.05 | 0.08 | 0.06 | 0.17 (0.02) | 0.09 |

| Rise | Simulation E_2 | Simulation E_3 | Simulation E_4 | Reference DNA | Classical B-type DNA |
| --- | --- | --- | --- | --- | --- |
| Step 5 | 3.61 | 3.73 | 3.64 | 3.83 (0.04) | 3.32 |
| Shift |  |  |  |  |  |
| Step 5 | 0.41 | 0.13 | 0.29 | 0.28 (0.10) | 0.00 |
| Step 6 | -0.21 | -0.18 | -0.20 | -0.26 (0.03) | 0.00 |
| Slide |  |  |  |  |  |
| Step 6 | -0.70 | -0.61 | -0.71 | -0.85 (0.06) | 0.53 |
| Tilt |  |  |  |  |  |
| Step 6 | -1.75 | -2.14 | -1.88 | -3.42 (0.33) | 0.01 |

(a)


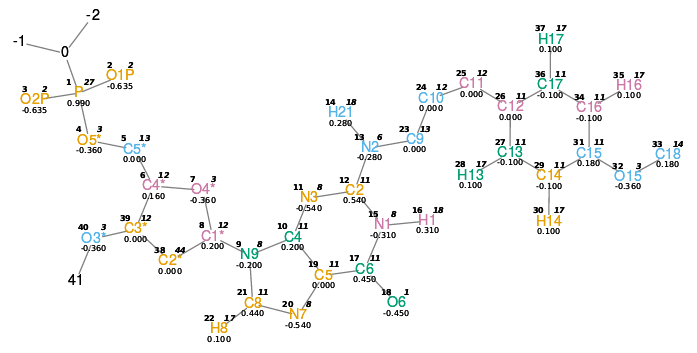


(b)


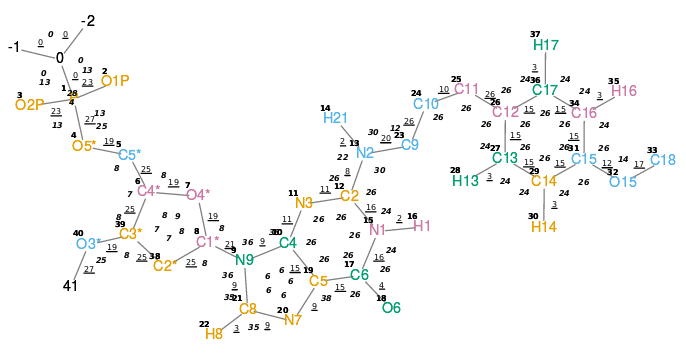


**Fig.S 1** Molecular topology building block for the E-3’-*N^2^*-dG DNA adduct. a) Indication of atom types (integer atomtype code; right hand superscripts) and partial charges (subscripts). Atoms with the same color form a single charge group. b) Indication of bond-vibration types (underlined values) and angle-bending types (bold italic values). All force field parameters according to the GROMOS 45A4 parameter set


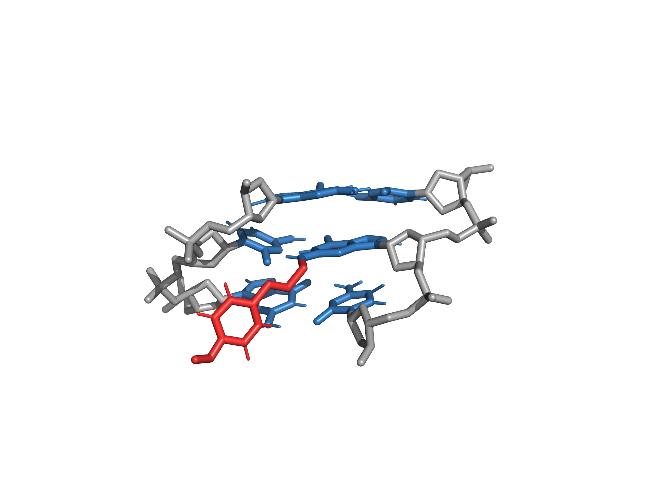

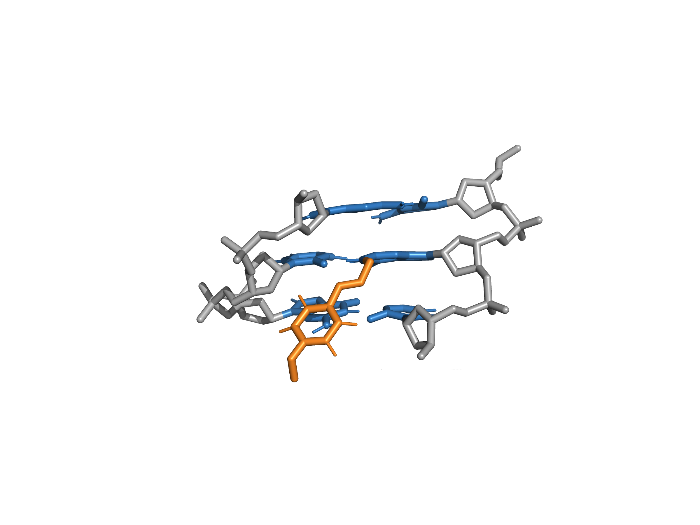

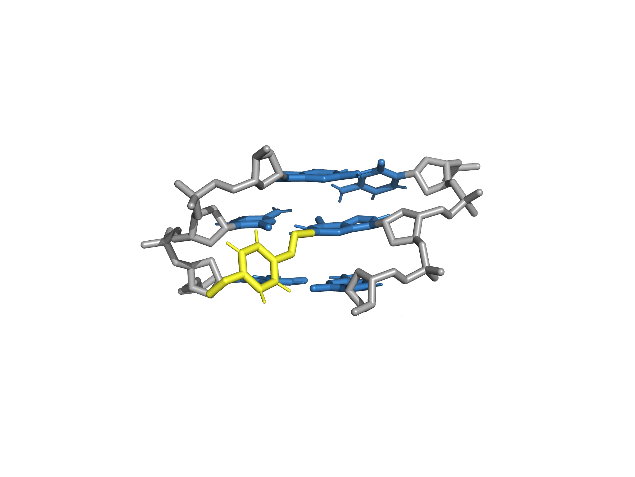

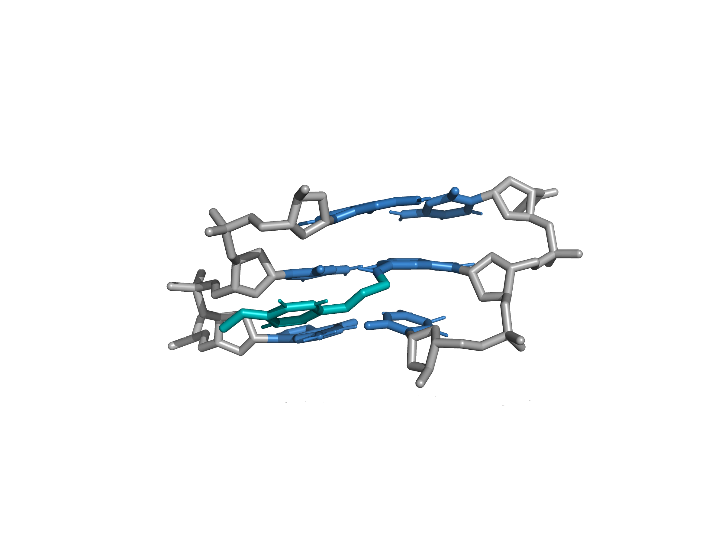


Simulation E_1

Simulation E_2

Simulation E_3

Simulation E_4

**Fig.S 2** Initial structures of simulations starting from four conformations of an E-3’-*N^2^*-dG adduct in the central 3-mer (C5-G6*-C7) viewing into the minor groove

Simulation E_1

Simulation E_2

Simulation E_3

Simulation E_4


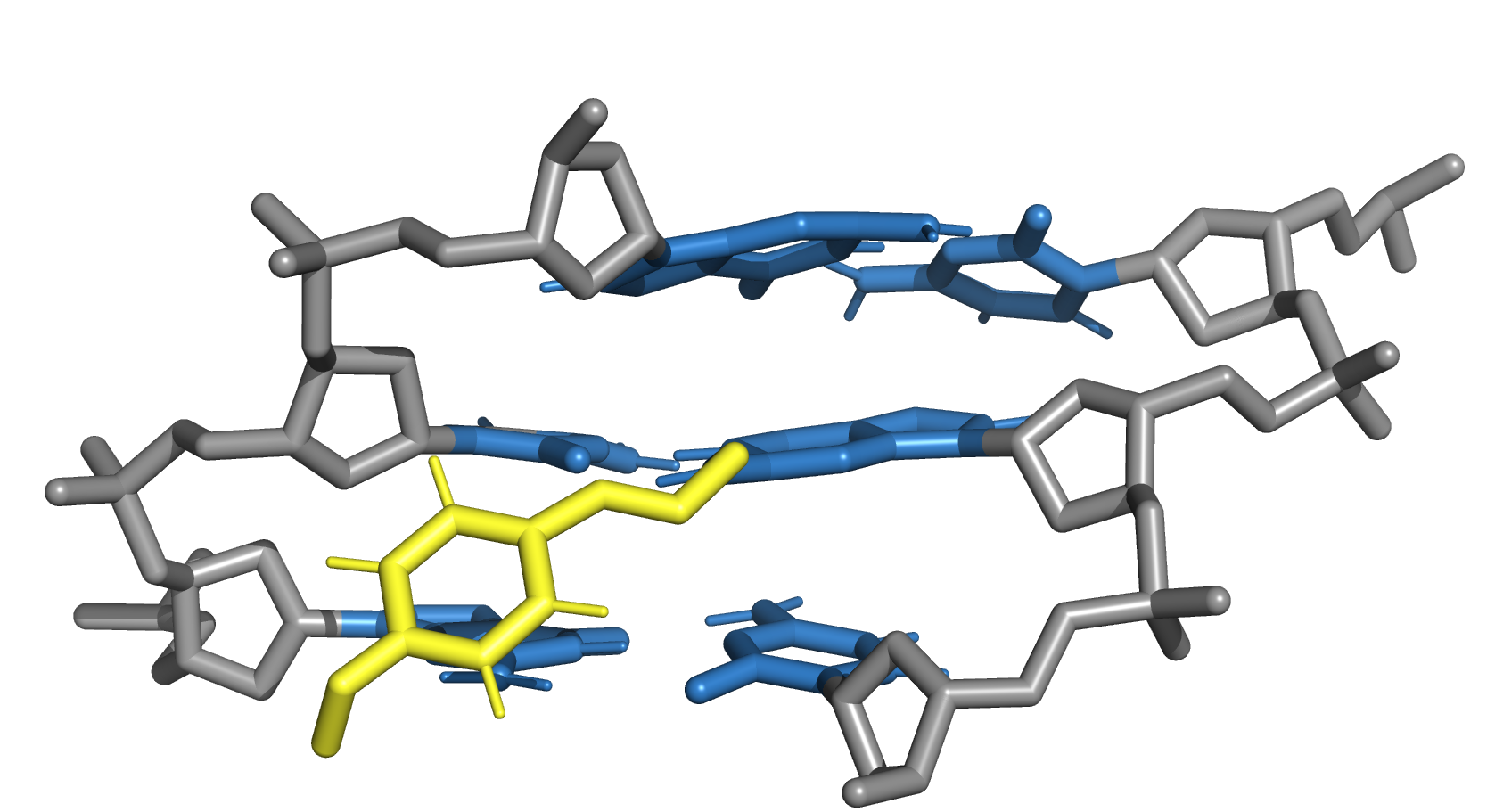

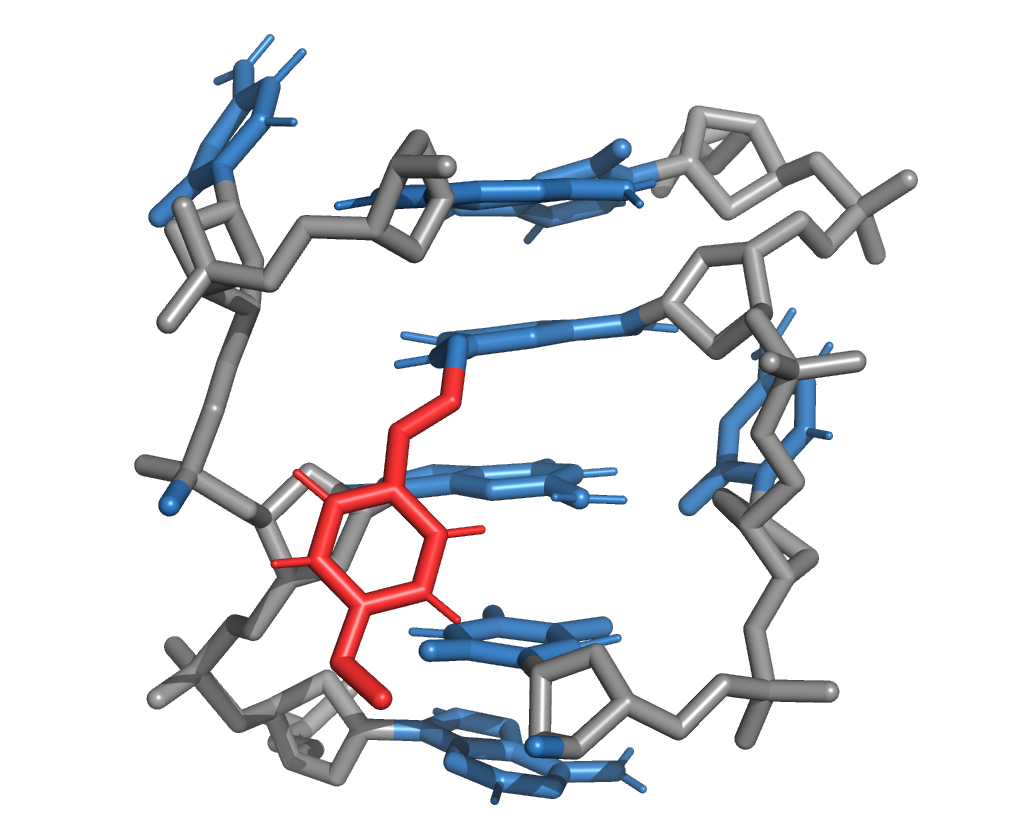

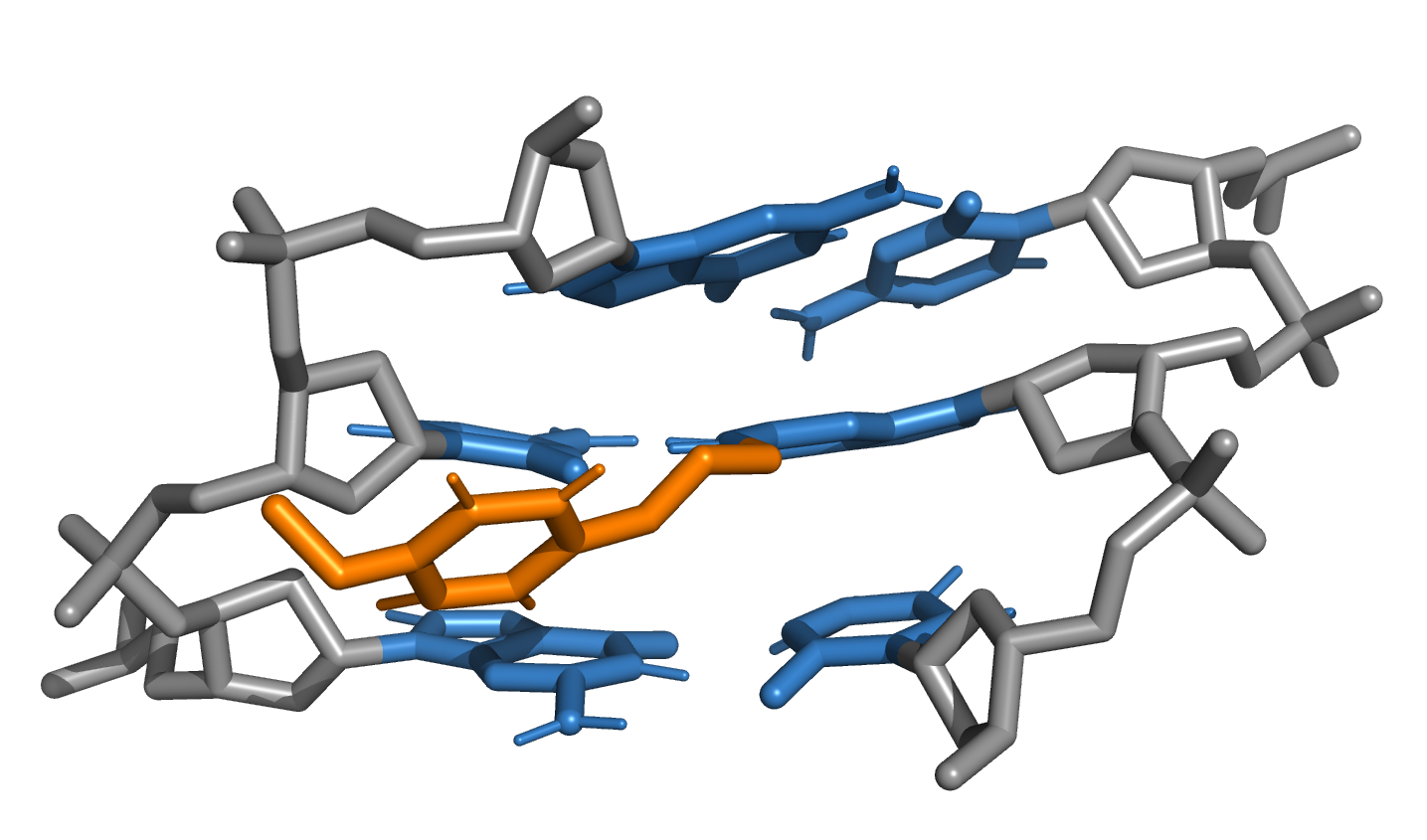

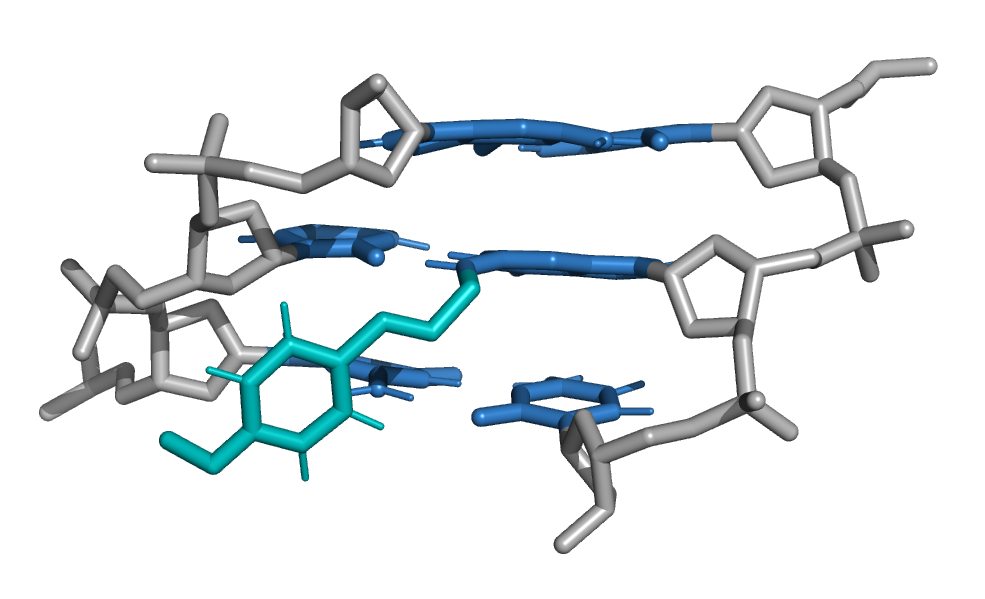


**Fig.S 3** Final state structures of simulations starting from four conformations of an E-3’-N^2^-dG adduct in the central 3-mer (C5-G6*-C7) for E_2-4 and the central 4-mer (C5-G6*-C7-T8) for E_1 viewing into the minor groove


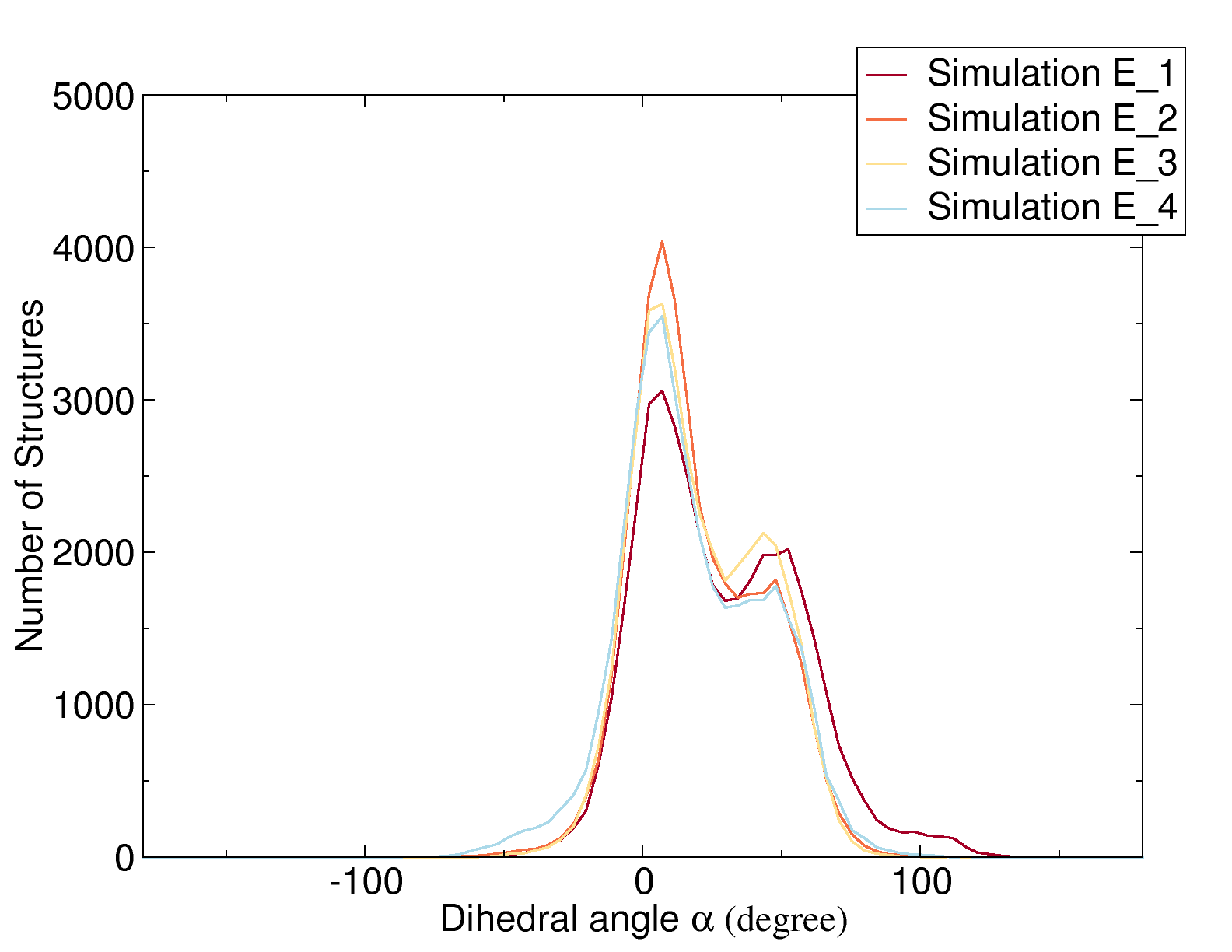

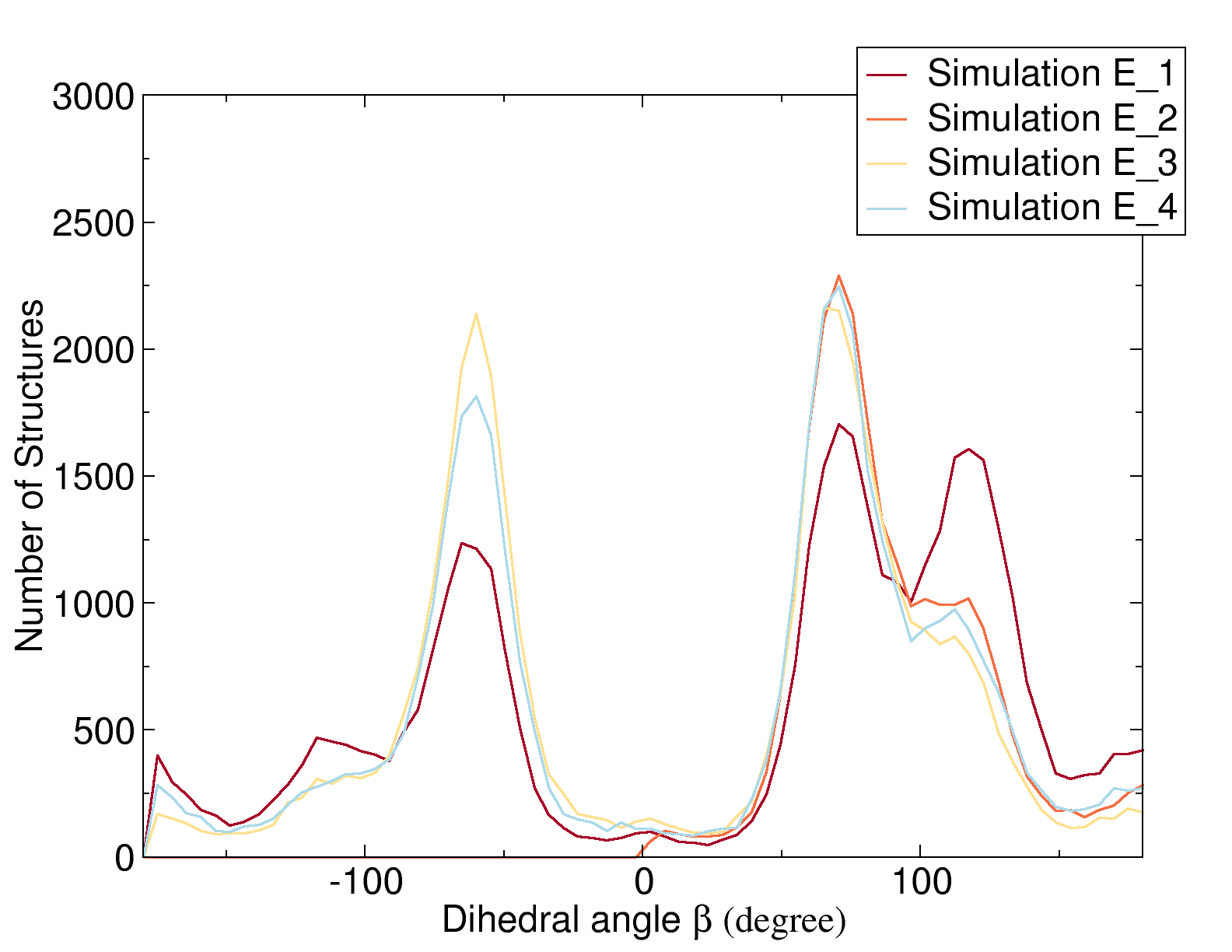


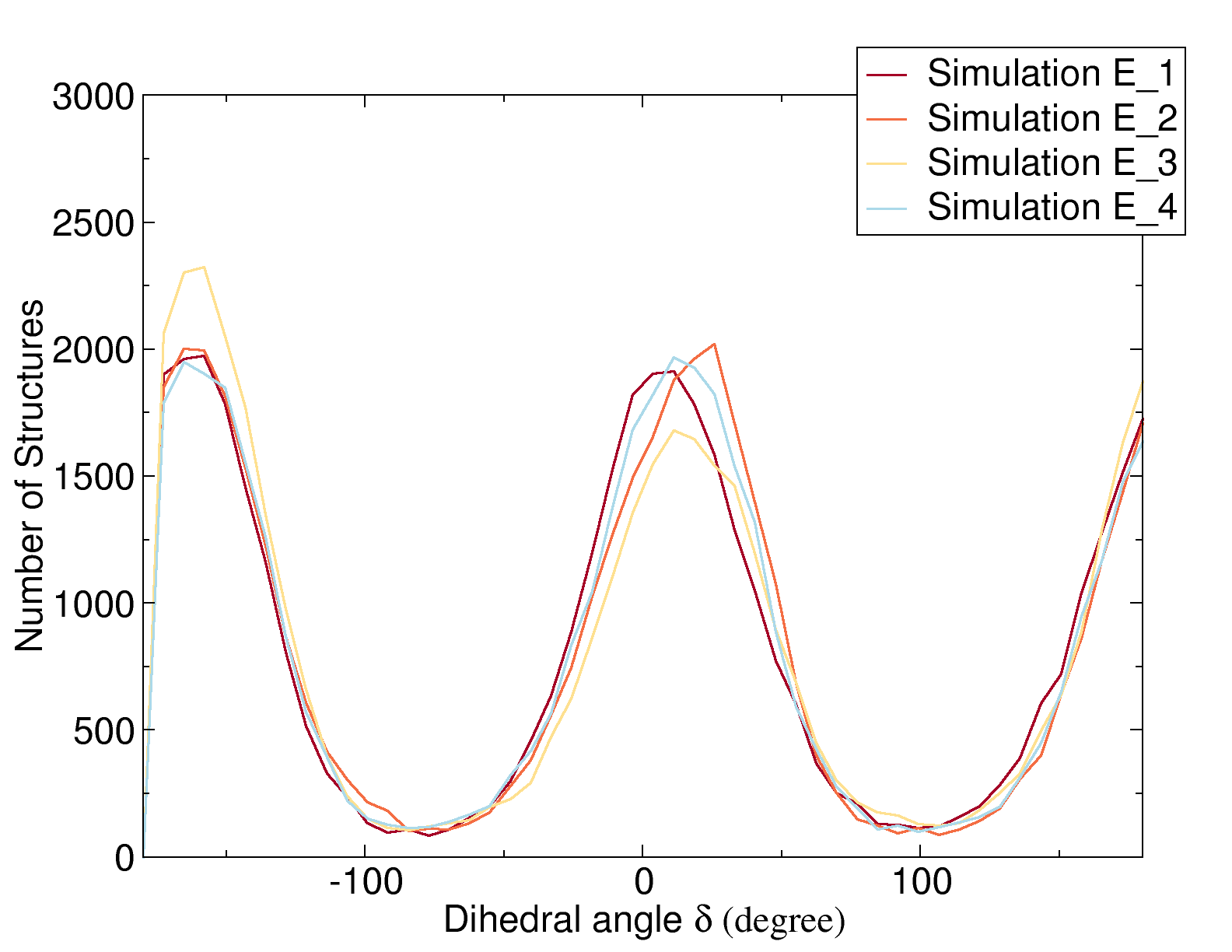


**Fig.S 4** Population distribution of torsion angle α, β and δ in different simulations


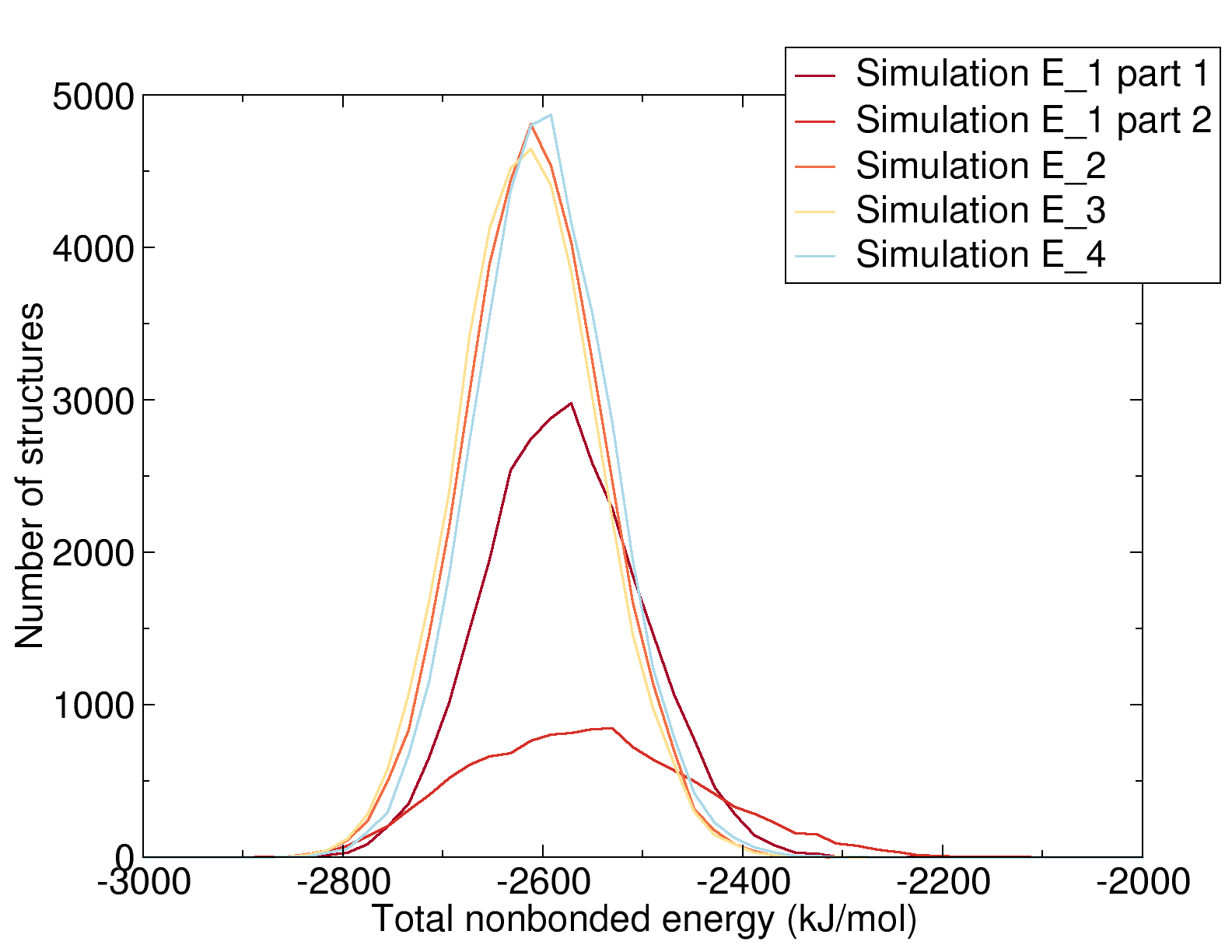

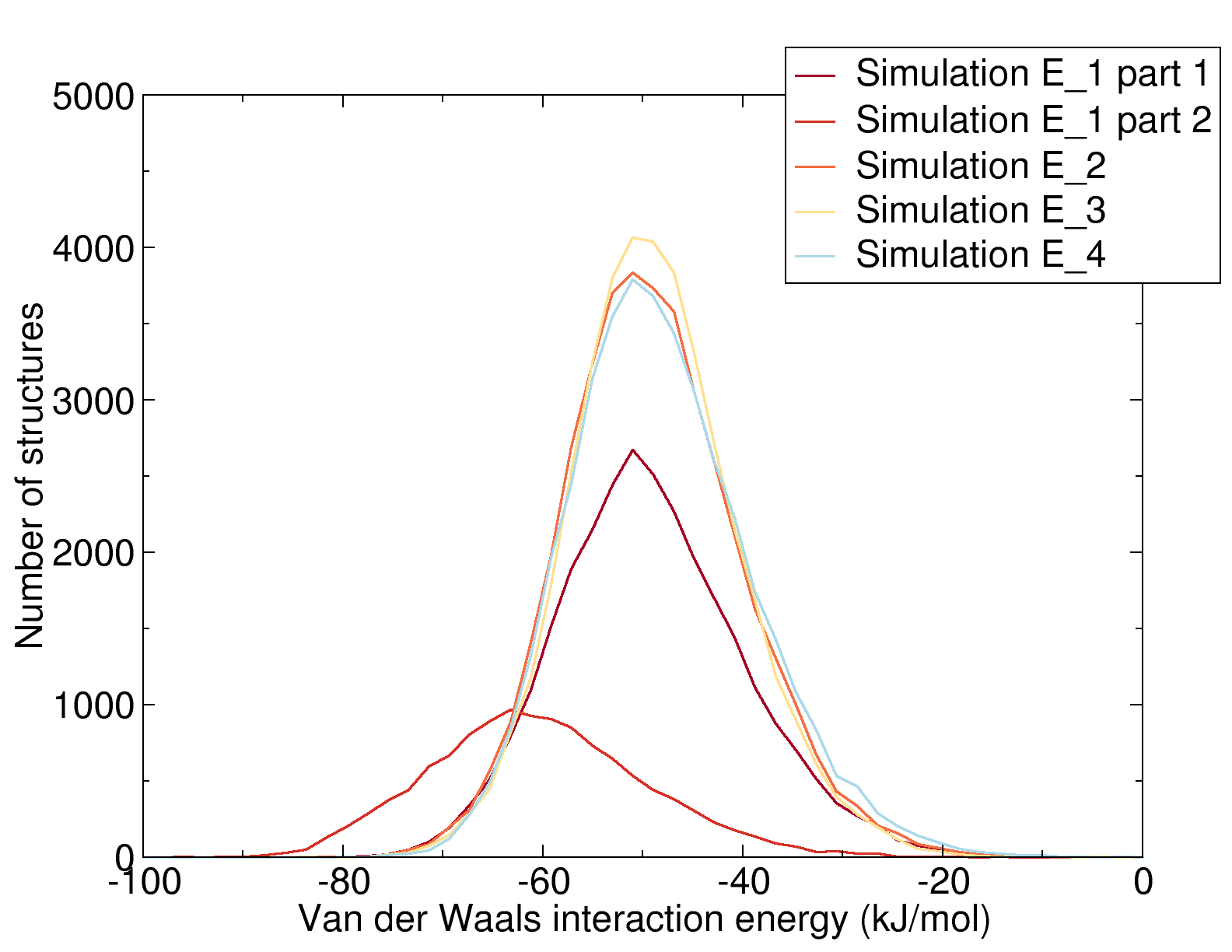


(b)

(a)

**Fig.S 5** Population distribution of the non-bonded interaction energy (including Van der Waals interactions and electrostatic interactions) for the overall DNA helix excluding counter ions and solvent (a) and the population distribution of total van der Waals interaction energy between the estragole adduct residue and its surroundings (including ions) without solvent (b). Population distribution of simulation E_1 are divided into E_1 part 1 (0-14 ns) and E_1 part 2 (14-20 ns)


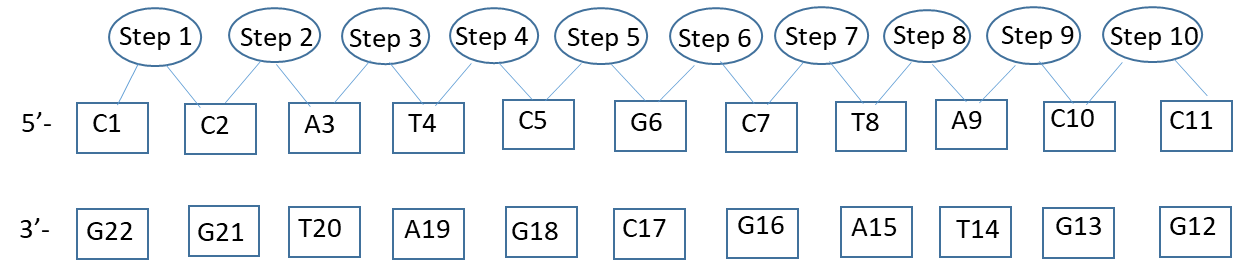


**Fig.S 6** Definition of base pair steps

(a)


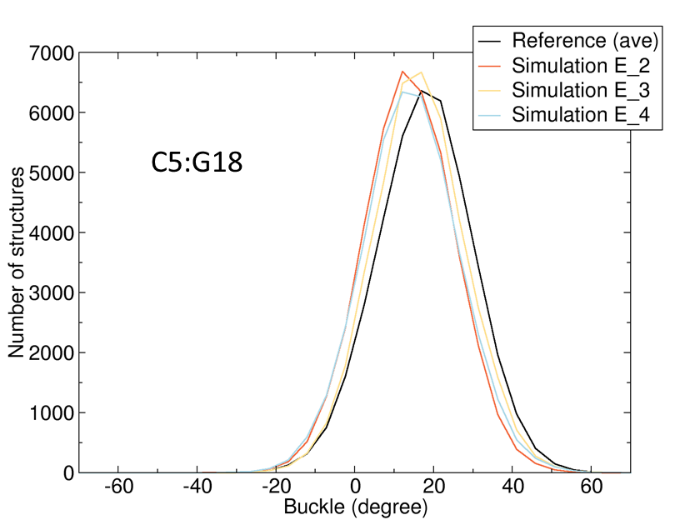

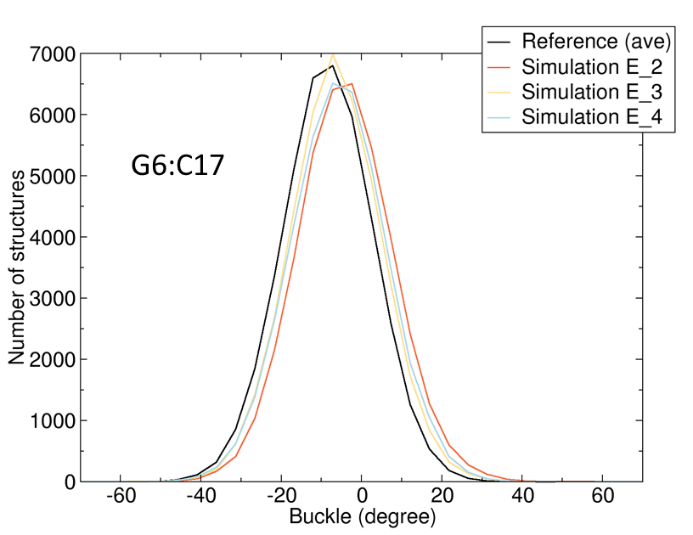


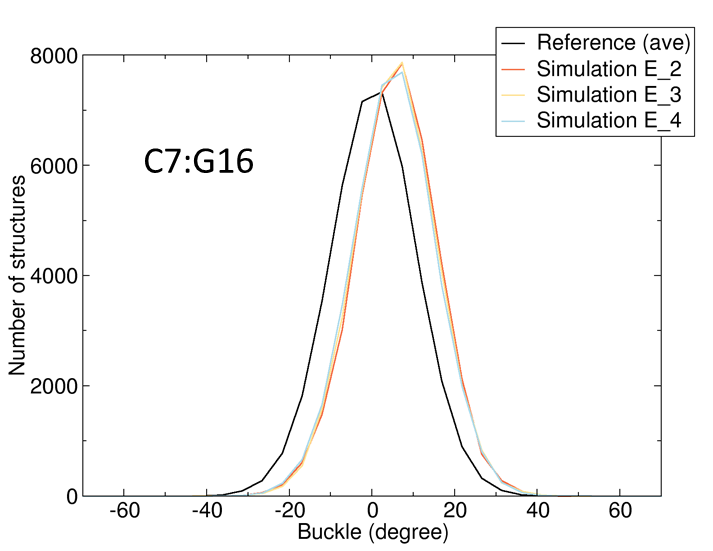


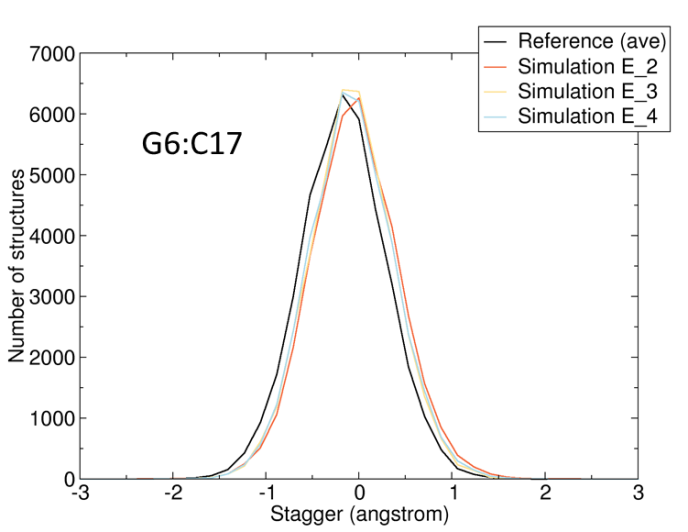

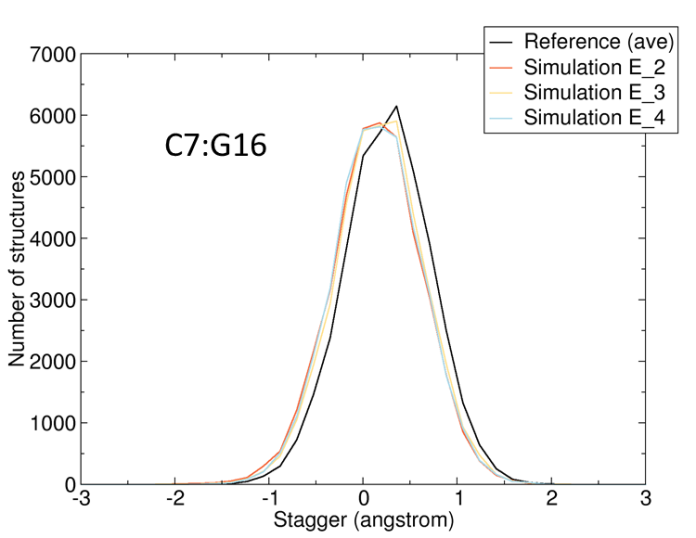


(b)


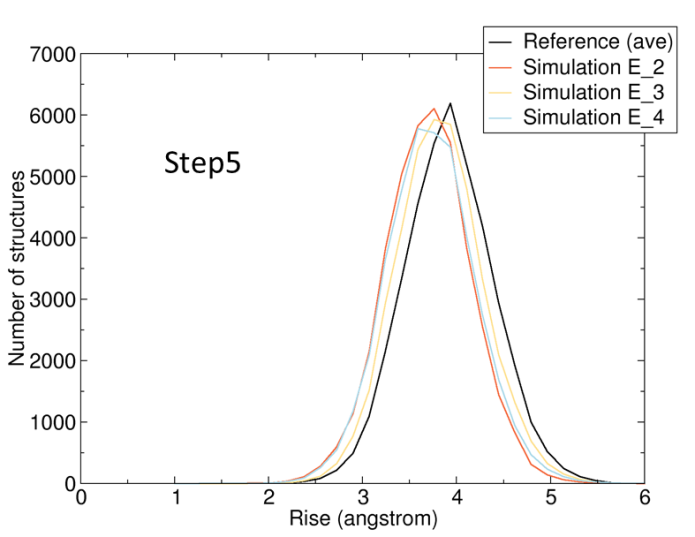

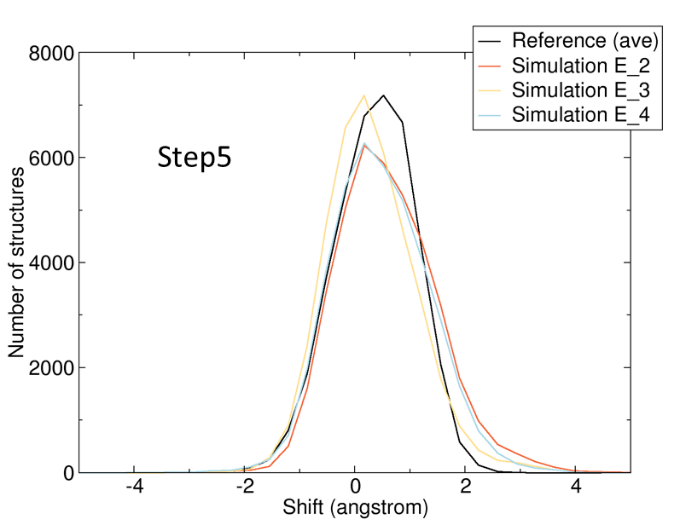


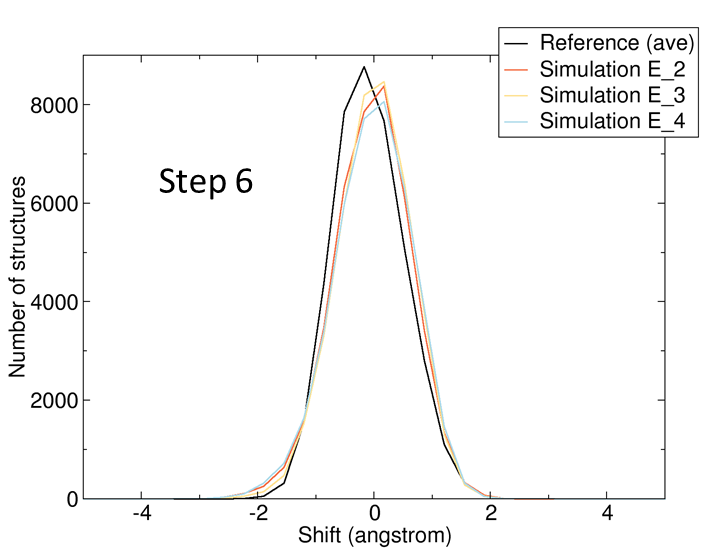

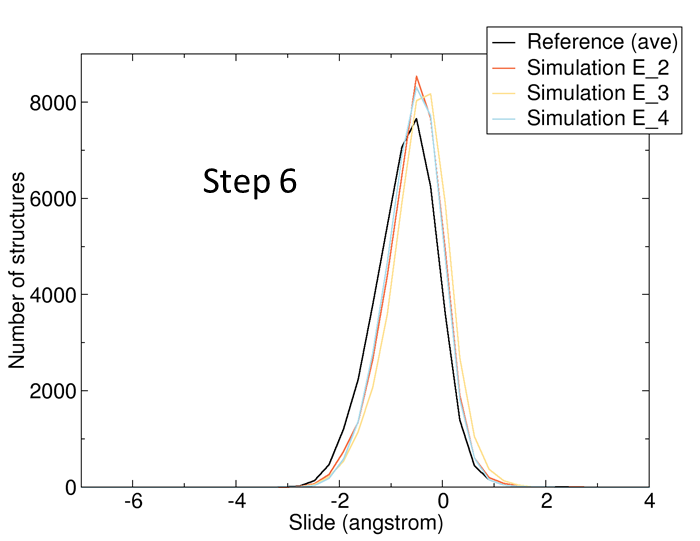


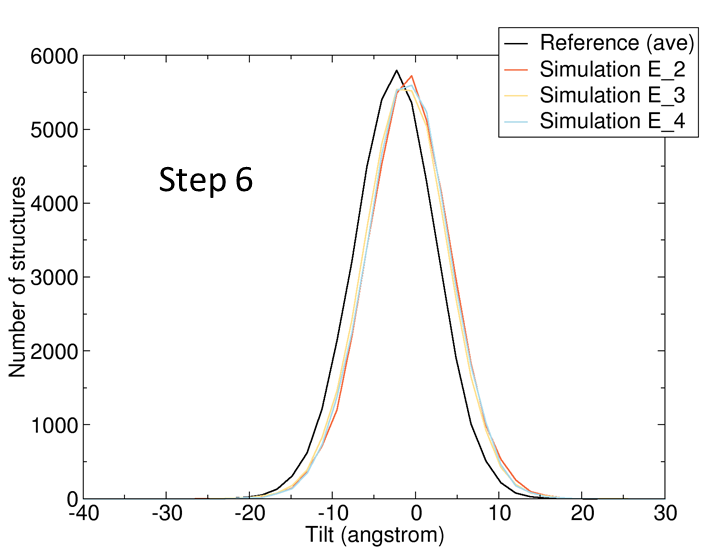


**Fig.S 7** Selected population distributions of duplex helicoidal parameters, (a) local base pair parameters (b) base pair step parameters. Only distributions with clear differences between modified DNA and reference DNA are shown

| (a) | 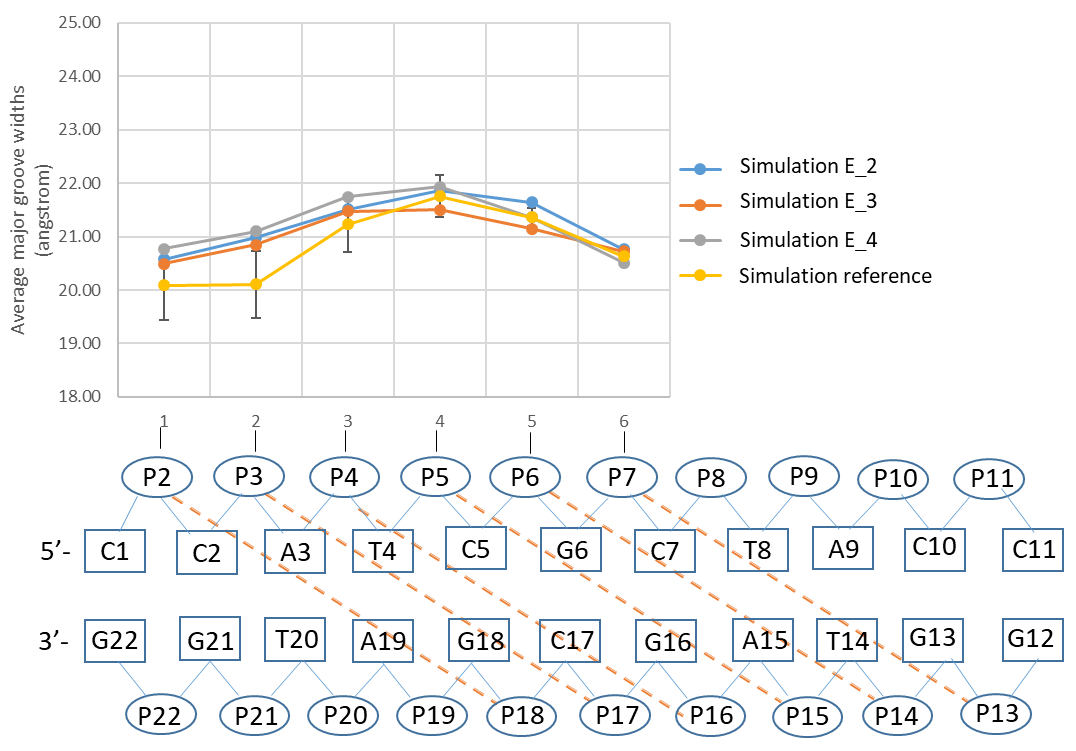 |
| --- | --- |
| (b) | 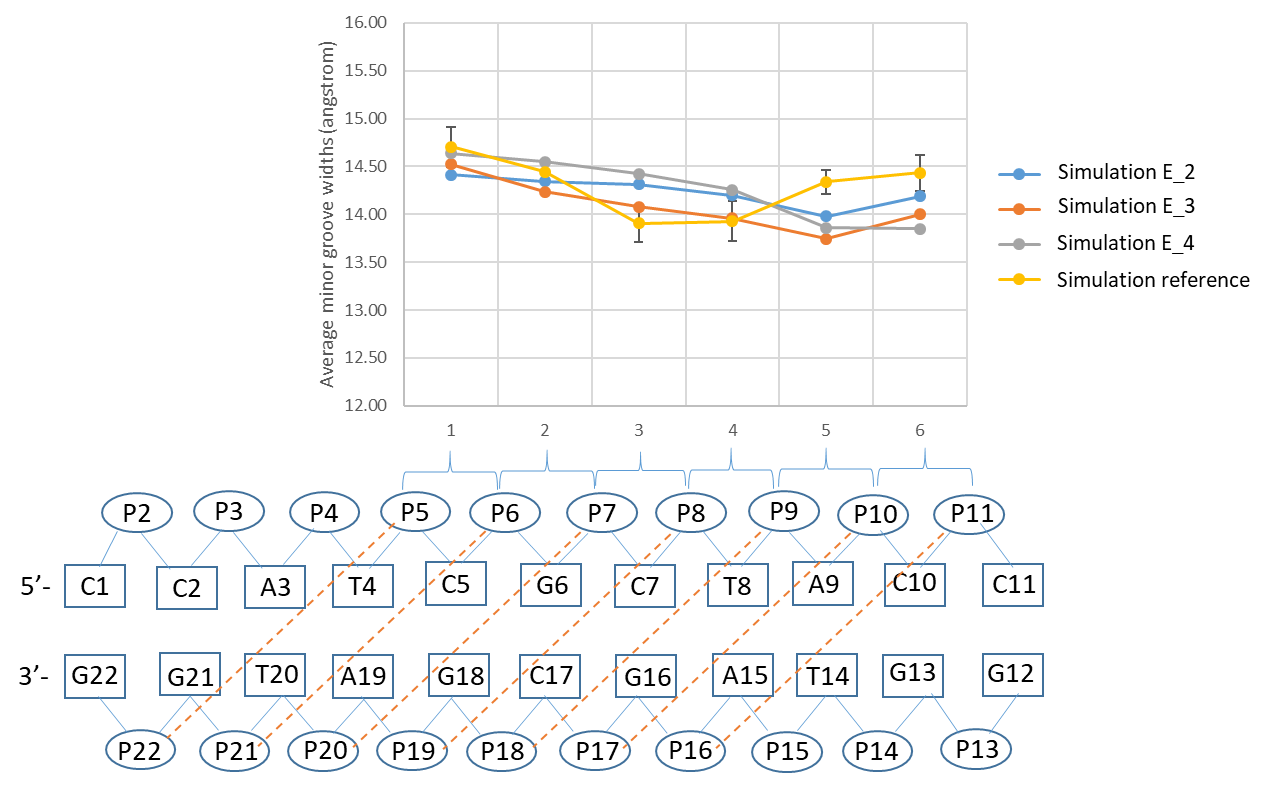  **Fig.S 8** Average major groove widths (a) and minor groove widths (b) for three conformations of estragole modified DNA and reference DNA duplex (with standard deviation). The major groove widths are measured directly between phosphorus- phosphorus atom. The minor groove widths are measured as the average of two adjacent phosphorus- phosphorus distances |
